# New evidence from exceptionally “well-preserved” specimens sheds light on the structure of the ammonite brachial crown

**DOI:** 10.1038/s41598-021-89998-4

**Published:** 2021-06-04

**Authors:** C. P. A. Smith, N. H. Landman, J. Bardin, I. Kruta

**Affiliations:** 1 Biogéosciences, UMR 6282, Université Bourgogne Franche-Comté-CNRS-EPHE, 6 boulevard Gabriel, 21000 Dijon, France; 2 Division of Paleontology (Invertebrates), American Museum of Natural History, Central Park West at 79th Street, New York, NY 10024 USA; 3 CR2P – Centre de Recherche en Paléontologie, Paris, UMR 7207, Sorbonne Université-MNHN-CNRS, 4 place Jussieu, 75005 Paris, France

**Keywords:** Palaeontology, Evolutionary ecology, Palaeoecology

## Abstract

Ammonite soft body remains are rarely preserved. One of the biggest enigmas is the morphology of the ammonite brachial crown that has, up till now, never been recovered. Recently, mysterious hook-like structures have been reported in multiple specimens of Scaphitidae, a large family of heteromorph Late Cretaceous ammonites. A previous examination of these structures revealed that they belong to the ammonites. Their nature, however, remained elusive. Here, we exploit tomographic data to study their arrangement in space in order to clarify this matter. After using topological data analyses and comparing their morphology, number, and distribution to other known cephalopod structures, in both extant and extinct taxa, we conclude that these hook-like structures represent part of the brachial crown armature. Therefore, it appears that there are at least three independent evolutionary origins of hooks: in belemnoids, oegospids, and now in ammonites. Finally, we propose for the first time a hypothetical reconstruction of an ammonite brachial crown.

## Introduction

Ammonites are an abundant and iconic group of extinct marine organisms. Although they are ubiquitous in the fossil record, the anatomy of their soft body is unfortunately very poorly known, hindering our knowledge of their paleoecology and paleobiology. One of the biggest uncertainties involves the morphology of their brachial crown. According to phylogenetic bracketing, it is generally assumed that they had ten arms^1,2^. However, no remains of arms or arm structures have ever been discovered in ammonites, not even when internal organs are preserved^3^. This is most probably due to the retraction of the arms into the body chamber post-mortem^1^, and/or the poor preservation potential of the arms’ soft tissue^4,5^. Additionally, ammonites are thought to have been preyed upon by many predators^6–9^, and even possibly by other ammonites^10^, further reducing the probability of preserving soft tissues.

On the other hand, arm crowns are well documented in fossil coleoids through the presence of sclerotized arm structures such as hooks, most often isolated^11–13^, but occasionally still articulated^14–20^ and/or associated with soft tissue remains^21,22^. Indeed, coleoid hook-like structures are reported in extant as well as in fossil coleoids since the Carboniferous^23,24^. The hooks in these coleoids (only present today in a few families of the order Oegopsida) differ in morphology, possibly implying that cephalopod hook-like structures appeared multiple times during the history of the group^25^. As a result, they are considered convergent acquisitions^23,26–28^. Therefore, it is essential to compare any fossilized structures in ammonites to those in both fossil and modern cephalopods.

**Figure 1:**
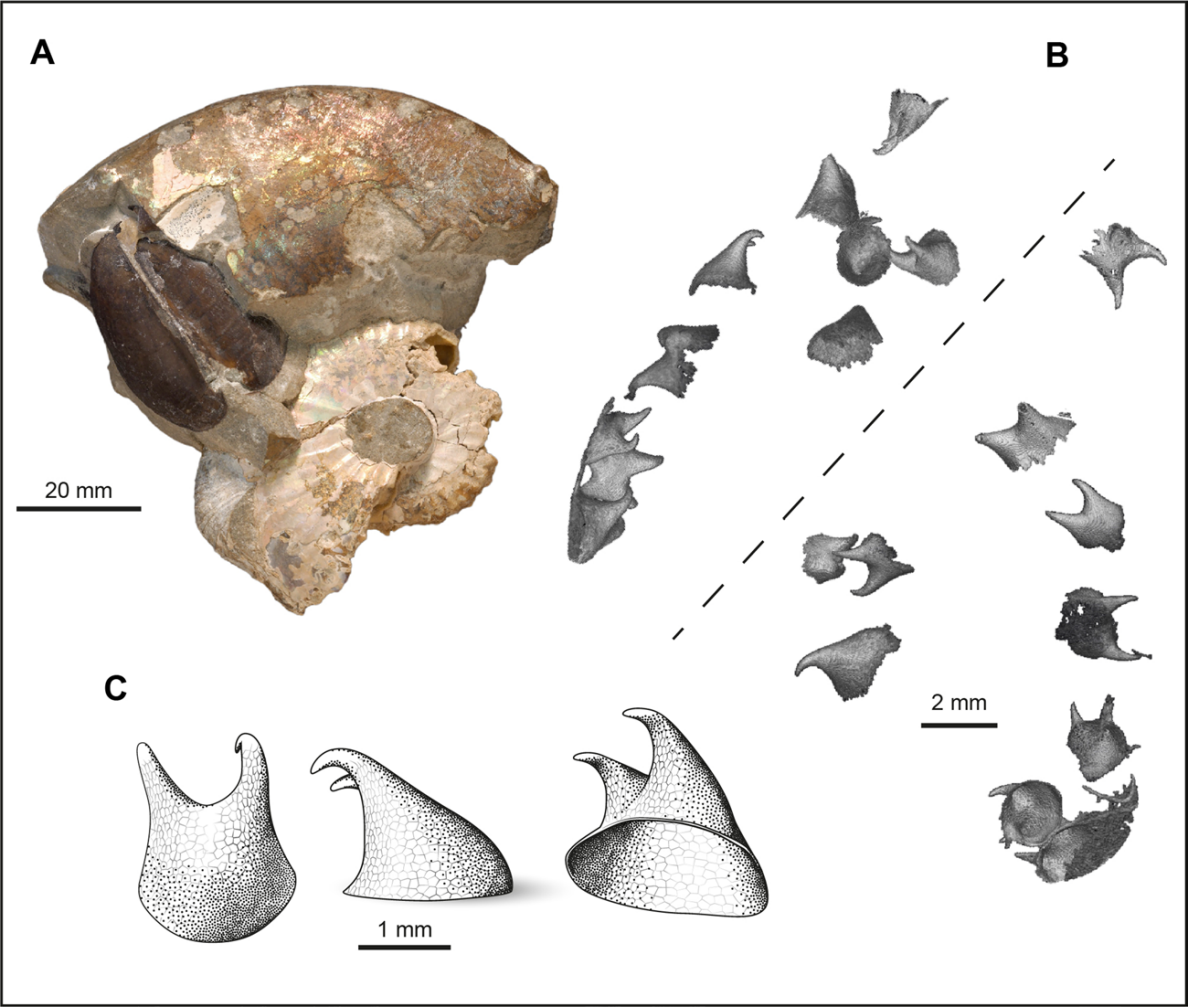
*Hoploscaphites* hooks. (**A**) *H. nicolletii*, AMNH 51333. Part of the phragmocone and most of the body chamber is preserved, with the jaw still *in-situ* testifying to the exceptional preservation. (**B**) Illustration of all the hooks uncovered in AMNH 51333 viewed from two different angles separated by the dashed line. The hooks have been reconstructed after segmentation using VGStudio MAX 3.0. (**C**) Illustrative drawing of *Hoploscaphites* hooks based on Landman et al.^29^ and the 3D rendering of the structures, drawing by A. Lethiers (CR2P).

In the last few decades, enigmatic hook-like structures have been discovered in multiple specimens of Late Cretaceous ammonites of the family Scaphitidae, a large group of heteromorph ammonites. They were first described by Landman and Waage^29^ who reported them in numerous specimens of *Hoploscaphites* from the Maastrichtian Fox Hills Formation of South Dakota (Fig. 1). At the time, the authors raised several questions regarding the nature of these structures: (1) Do they belong to the ammonite or are they the remains of some other organism? (2) If they belong to the ammonite, are they radular elements? (3) If not, what are they?

Kennedy et al.^30^ later described similar structures in *Rhaeboceras*, another member of the Scaphitidae, from the Campanian Bearpaw Shale of Montana. Based on the location of the structures (in the body chamber), they argued that these structures belonged to the ammonites and interpreted them as radular elements. They did, however, express reservations about such an interpretation because of the unusually large size of the structures (approximately 50% of the length of the upper jaws) and the important morphological differences with other known radular elements.

**Figure 2:**
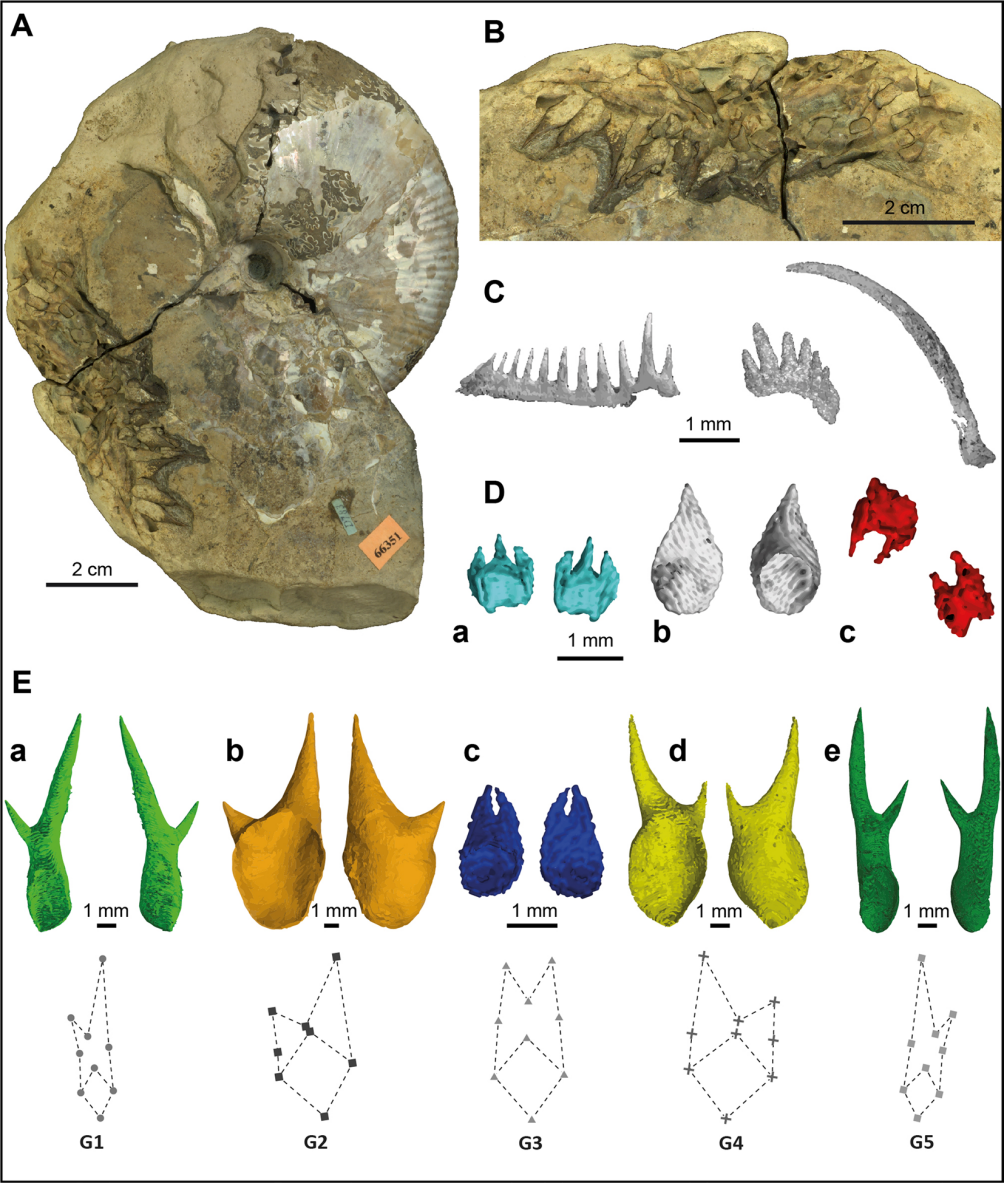
*Rhaeboceras halli* hooks. (**A**) *R. halli*, AMNH 66351 with hooks occurring at the edge of the body chamber. (**B**) Close-up view of the hooks in AMNH 66351. (**C**) *R. halli* radular teeth identified by Kruta et al.^31^; from left to right: second lateral tooth, first lateral tooth and marginal tooth. (**D**) & (**E**) 3D rendering (VGStudio MAX 3.0.) of the structures identified in Kruta et al.^32^. (**D**) (a) tricuspid, (b) unicuspid, (c) very small bicuspid structures. (**E**) The five main bicuspid morphotypes and their typical shape.

Their concerns were justified, as Kruta et al.^31^ rejected the radular interpretation after discovering evidence of radulae in three specimens of *Rhaeboceras halli*. The morphology of the radular teeth reported was consistent with that of radular teeth known from other aptychophoran ammonites (Fig. 2C) and was completely unlike the hook-like structures previously described. These hook-like structures have now been documented in approximately 50 specimens of *Rhaeboceras halli* and closely related species. The study of these structures is complicated, however, by the fact that most of them are embedded in the sedimentary matrix filling the body chamber. Using high resolution X-ray imaging, Kruta et al.^32^ managed to capture the morphology of the structures in several specimens. They documented a large number of structures (as many as 171 in a single specimen) and described them as hook-like structures, categorizing them according to morphotype. They also emphasized that the size and shape of the structures were inconsistent with the radular tooth hypothesis, rejecting it once and for all. Instead, they suggested a possible brachial crown interpretation, leaving open the path for future investigation.

The present work further investigates the nature of theses mysterious hook-like structures in *Rhaeboceras halli* by studying their arrangement in space and comparing them with other known cephalopod structures. To accomplish this, we used high resolution X-ray imaging data to obtain complete 3-D images of all of the structures and their distribution in space in several specimens. Applying statistical analyses, including persistent homology (i.e., a type of topological data analysis that consists in assessing topological features from a data set based on the proximity of the points in space; for more detail see Supplementary Material section “presentation of persistent homology”), we explore the distribution of the structures in each specimen with an emphasis on the spatial distribution of the various morphotypes. Several common patterns emerged allowing us to reconstruct the arrangement of the hooks on the arms. This leads, for the first time, to an interpretation of the morphology of the brachial crown in ammonites.

## Results

### Description of the hooks

The hooks in Scaphitidae are thin-walled (150 µm thick in *Rhaeboceras halli*), hollow structures (Figs. 1 and 2) that are generally bicuspid although in *R. halli*, a few (2%) are tricuspid (Fig. 2D–a), rounded or unicuspid (Fig. 2D–b). The base always exhibits a rather large opening (Figs. 1C and 2D, E) that may be related to soft tissue insertion, as in coleoid hooks^23,33^. In *Hoploscaphites*, the hooks are slightly curved towards the end of their equally short cusps, have a wide round opening (2–5 mm in diameter), and do not vary in size or shape^29^ (Fig. 1). In contrast, the hooks in *R.halli* tend to be straight with an oval slanted opening at their base and show a broad range of morphologies (Fig. 2D, E). Therefore, we use the designation “hook” as a general term for any pointy structure despite the fact that these structures do not necessarily curve backward. Kruta et al.^32^ divided the hooks into five major bicuspid morphotypes. Morphotype 1 (G1) is elongate with the right cusp longer than the left one (Fig. 2E–a). Morphotype 2 (G2) is large and wide with the right cusp longer than the left one (Fig. 2E–b). Morphotype 3 (G3) is slightly smaller than the other morphotypes with both cusps of equal size (Fig. 2E–c). Morphotype 4 (G4) is large and wide with the left cusp longer than the right one (Fig. 2E–d). Morphotype 5 (G5) is elongate with the left cusp longer than the right one (Fig. 2E–e). The authors emphasized that morphotypes 1 and 5, and 2 and 4, were mirror images of each other, respectively. They also described several very small bicuspid hooks (Fig. 2D–c) with cusps subequal in size.

After fully reconstructing AMNH 95795, 122 hooks were reported; all of them are attributable to one of the 9 morphotypes (5 major bicuspid morphotypes, the very small bicuspid morphotype, and the tricuspid, unicuspid and rounded morphotype) described in Kruta et al.^32^. Many hooks were also uncovered in AMNH 160989 but because of their chaotic distribution in the body chamber, we did not include this specimen in our study (the number of hooks of each morphotype for each specimen is available in Supplementary Table S1).

### Position in the body Chamber

In all the scanned specimens (8 specimens hosting hooks), the hooks are grouped in clusters. Therefore, we assume that the hooks in many non-scanned specimens are also grouped in clusters. Thus, even if only a part of the cluster is visible, it marks the position of the entire assemblage. The hooks always occur in the body chamber. The side of the body chamber on which the hooks occur, however, varies from one individual to another and there seems to be no pattern in their distribution as they are on the right flank, left flank, or venter; they can be in the middle or posterior part of the body chamber, but rarely in the anterior part (Table S2). In specimens with jaws preserved in-situ (8 specimens; 28% of the specimens), the hooks are located beneath or behind the jaws but never inside them. Kennedy et al.^30^ reported one specimen in which the hooks appear to occur inside the jaws. However, after re-examining this specimen, we observed that some of the hooks actually point out of the jaw. We conclude that these hooks were not originally located in the jaw, but instead, left impressions on the jaw following the death of the animal, either due to gravitational or compressional processes during fossilisation.

### Hook distribution

**Figure 3:**
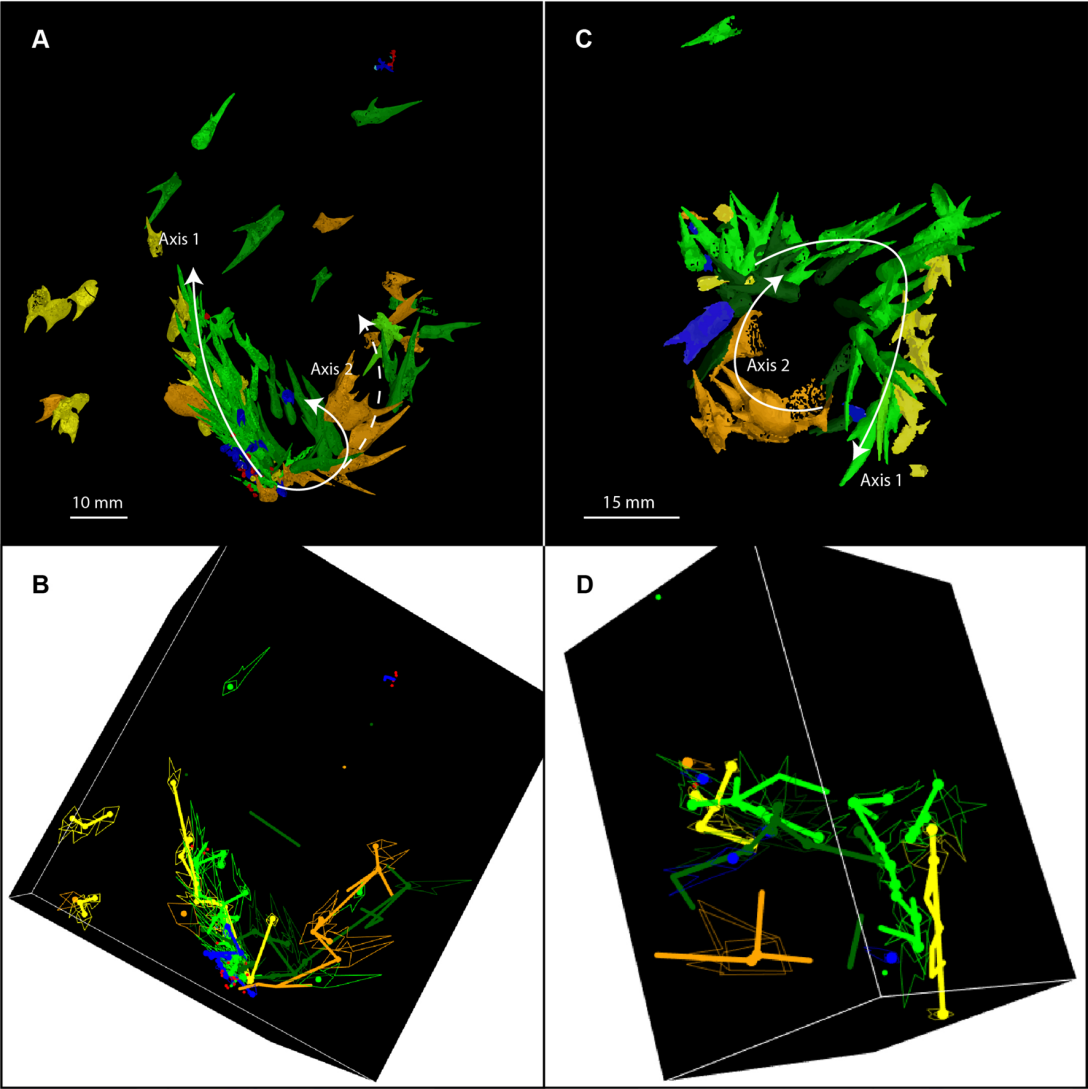
Distribution of the hooks *in-situ* in *Rhaeboceras halli*. (**A**) & (**B**) AMNH 66350. (**C**) & (**D**) AMNH 66433. (**A**) & (**C**) 3D rendering (VGStudio MAX 3.0.) of the structures preserved in the body chamber. (**B**) & (**D**) Simplified representation of the distribution of the hooks in space (R software, package rgl^65^). The thick lines represent the links between the hooks according to morphotype, based on the persistent homology analysis of the centroid position of their opening. Only the strongest and best integrated links are shown. The white arrows indicate the two suspected axes. Additional animated figure is available in the corresponding supplementary “.gif” document and interactive plot is available in the corresponding supplementary “.html” document.

Our results reveal that not only do the hooks always occur together inside the body chamber, but they are also arranged by morphogroup. Based on the distances between hook centroids, we determined that the nearest neighbour of each hook is most often a hook of the same morphotype (Table 1; detail for each specimen in Supplementary Table S3). The hooks are, thus, non-randomly distributed. The hooks of the same morphotype are also commonly aligned in longitudinal rows, either straight or in an arc, and are imbricated. This pattern is particularly well illustrated using the persistent homology analyses (Fig. 3). When the hooks are arranged in a straight line, all of the cusps point in one direction (Fig. 3A). When the hooks are arranged in an arc, the cusps point outward (Fig. 3A, C). These patterns are conspicuous in the best preserved specimens i.e., the specimens with the most hooks such as AMNH 66350 and 66433.

**Table 1:**
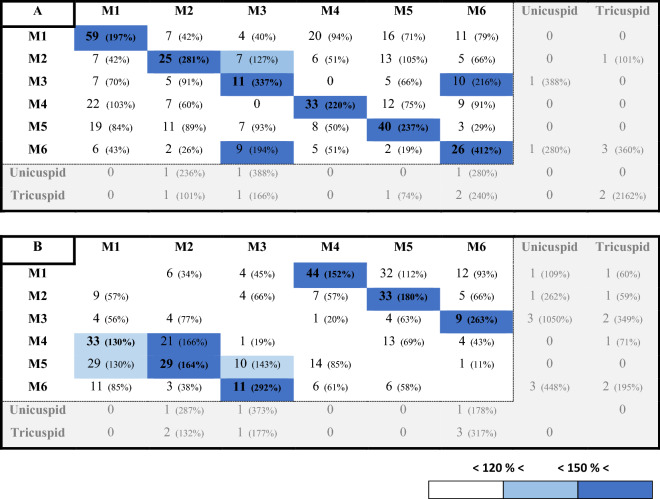
Summary of the nearest neighbour to each hook based on the centroid position of the hooks, according to morphotype and over all specimens: **A** With all the hooks. **B** Only taking into account hooks of a different morphotype, after excluding outliers. The colour scale is dependent on the counts, in comparison to what would have been expected if the hooks were randomly distributed in the body chambers. This does not apply to the unicuspid and tricuspid morphotypes due to too few representatives. The number in brackets represents the ratio observations/expected if randomly distributed. For detail per specimen, see Tables S3 & S4.

### Morphotype associations

We also noted associations between pairs of morphotypes (Table 1B). Morphotype 1 is most closely associated with morphotype 4 in four of the seven fully reconstructed specimens (AMNH 66350, 66433, 66434, and 95795; Table S4). In AMNH 66350 and AMNH 66433, the two morphotypes are aligned side by side, with the longest cusps next to each other (Fig. 3). In AMNH 66434 and 95795, although the distribution of the hooks seems a bit more chaotic, morphotypes 1 and 4 are still grouped together (Table S4; Fig. S2). In AMNH 66448, the two morphotypes are not touching but are distributed fairly close to each other along the same arc and in the same plane (Fig. S3A, B). In the two remaining specimens (Figs. S3 C, D and S4), very few (2 G1 and 2 G4 for AMNH 66351; 8 G1 and 7 G4 for AMNH 64405) of both morphotypes are present, which explains why the relationship between the two morphotypes is not apparent.

Morphotypes 2 and 5 are also associated with each other in five fully reconstructed specimens (AMNH 66350, 66433, 66434, 95795, and 66351; Table 1B). In AMNH 66350, these two morphotypes are arranged side by side, with the longest cusps next to each other, forming a second axis (Fig. 3A,B). In AMNH 66434, the two morphotypes are grouped together (Fig. S2A,B), and in AMNH 66433, they are distributed along the same arc (Fig. 3C,D). Most of the structures in AMNH 66351 are of morphotype 2 or 5 (19 out of the 26 attributed to a morphotype). In AMNH 95795, morphotypes 2 and 5 are arranged together and underneath morphotypes 1 and 4 (Fig. S2C,D). This pattern also seems to appear in AMNH 66448, but there are too few hooks of morphotype 5 to be sure (Fig. S3A,B). When smaller morphotypes (morphotype 3 and the very small hooks) are present in the same specimens (AMNH 66350, 66433, and 95795), they occur together at the base of the axis described by the other morphotypes (Figs. 3 and S2).

## Discussion

Our study of the hooks in *Rhaeboceras halli* confirms the existence of at least nine morphotypes, as previously demonstrated^32^. Morphotypes 1, 2, 4, and 5 are also visible at the surfaces of several non-scanned specimens and we suspect that the other morphotypes are also present in these specimens, but embedded in the matrix. A detailed study of the hooks in *Hoploscaphites* has not yet been performed. Nevertheless, it is evident that *Hoploscaphites* hooks (Fig. 1) are different and less variable in shape than those in *R. halli* (Fig. 2). Thus, the following discussion and interpretations remain, for now, mostly restricted to *R. halli*.

**Figure 4:**
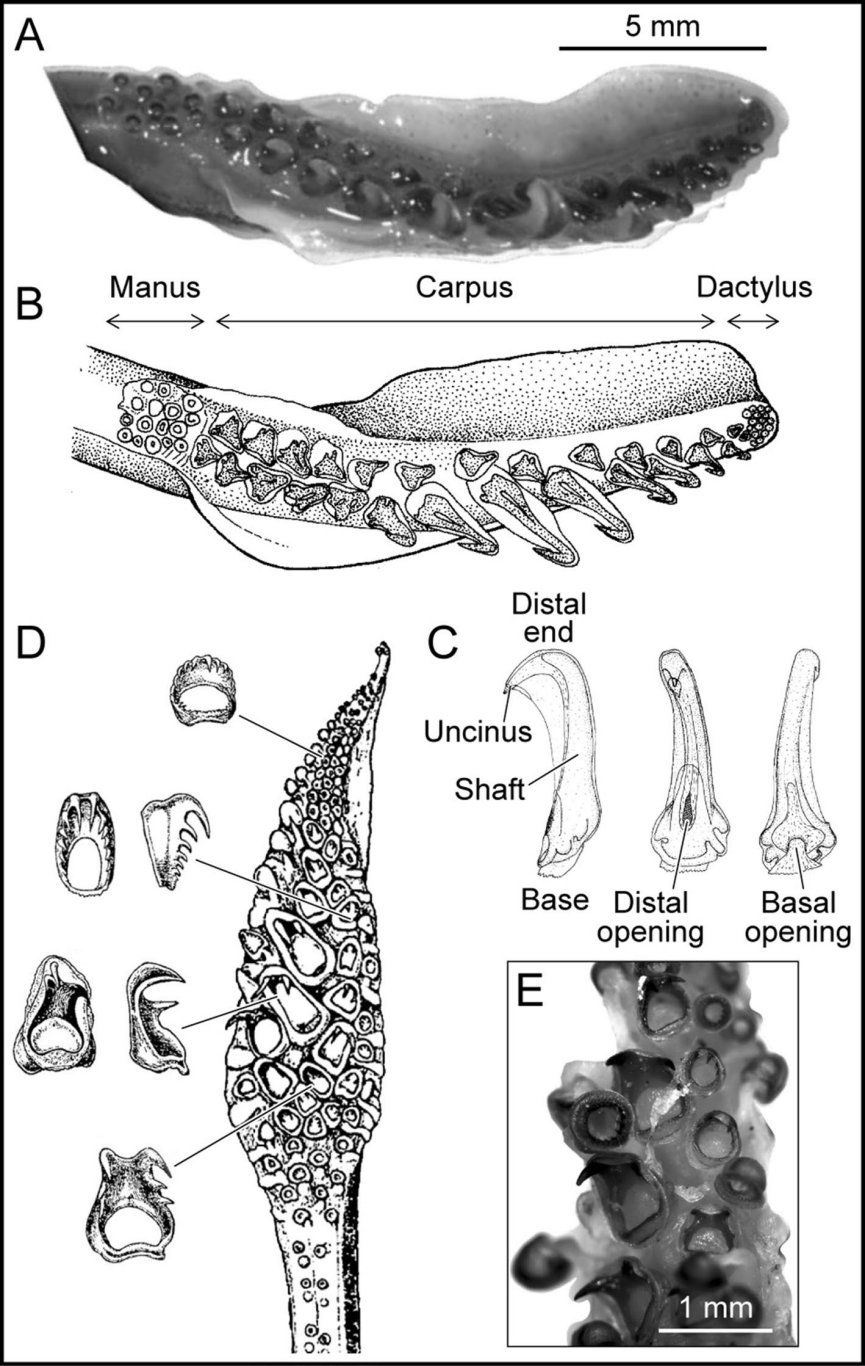
Examples of modern tentacular clubs and their armature. (**A**) *Onychoteutis banskii* left tentacular club, YPM 17906. (**B**) Sketch of a *Onychoteutis banskii* left tentacular club, modified from Roper et al.^66^. (**C** )Hook of *Onychoteuthis banskii* with soft tissues modified from Kulicki & Szaniawski^34^. From left to right: lateral view, outer side view and inner side view. (**D**) Sketch of a *Taonius pavo* right tentacular club with various modified suckers, after Sasaki^37^. (**E**) *Taonius pavo* left tentacular club manus, YPM 37340. In all specimens the base of the hooks is open on two sides (basal and distal.

**Figure 5:**
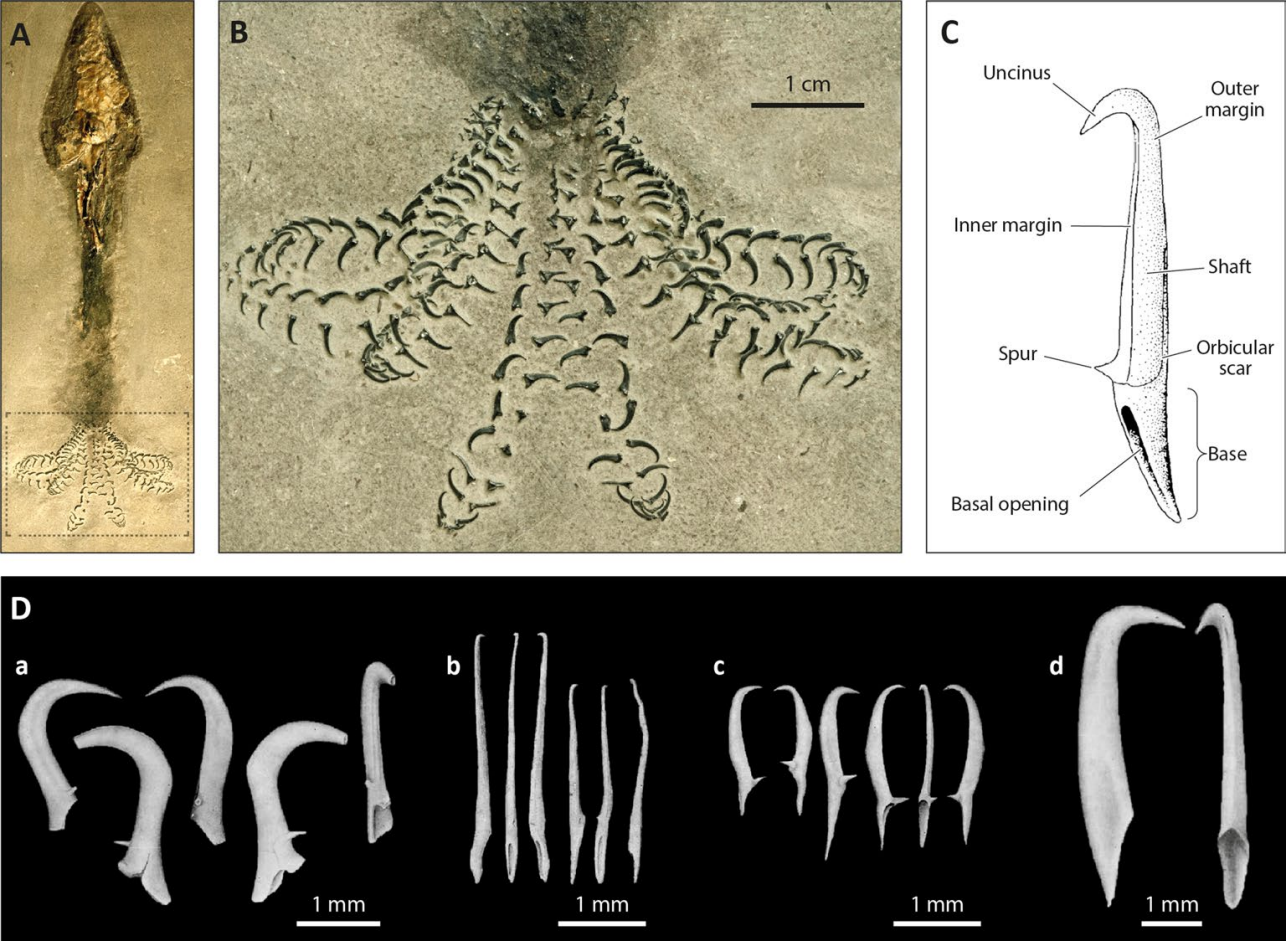
Examples of extinct belemnoid onychites. (**A**) Hook bearing belemnoid specimen, AMNH 046611. (**B**) Close up image of AMNH 046611, brachial crown. (**C**) Schematic drawing of a fossil arm hook with particular morphological elements and their terminology modified from Kulicki & Szaniawski^34^. (**D-**) Examples of different onychites identified by Kulicki & Szaniawski^34^ : *Falcuncus falcus* onychites (a); *Longuncus longus* onychites (b); *Paraglycerites gracilis* onychites (c); *Deinuncus brevirostris* onychites (d).

In modern cephalopods, hooks appear as brachial crown structures only among decabrachians in seven families of Oegopsida^25^ (Onychoteuthidae, Octopoteuthidae, Enoploteuthidae, Ancistrocheiridae, Pyroteuthidae, Gonatidae, and Cranchidae). The hooks are elongate, unicuspid, and curved, with a flared base and a double-sided opening (Fig. 4). Hooks in extinct cephalopods (onychites) such as Belemnitida, Donovaniconida, and Phragmoteuthida are also elongate, unicuspid, and curved. They differ from modern decabrachian hooks by often presenting a small spur on their left or right side and having only a single-sided slanted opening at their base^23,25,34^ (Fig. 5).

The morphology of the hooks in *Rhaeboceras halli* and *Hoploscaphites* is unique. The hooks are hollow with a well-defined bicuspid shape and a single-sided wide opening at their base. They do, however, share a few vague similarities with hooks of other cephalopods. The hooks in *R. halli* and *Hoploscaphites* fall in the same size range as those in modern decabrachians^35^ and belemnoids^12,36^. Morphotypes 2 and 4 in *R. halli* are similar in morphology to some of the bicuspid hooks of *Taonius pavo*^37,38^ (Fig. 4D,E). Morphotypes 1 and 5 in *R.halli* resemble onychites with well-developed lateral shafts such as the onychites of *Paraglycerites* (Fig. 5D–c).

In modern decabrachians, the hooks originate from modifications of the chitinous rings around the suckers^39,40^, hence their double sided opening. This contrasts with the single-sided opening of onychites. Engeser and Clarke^23^ argued that this difference between the hooks of modern decabrachians and belemnoids was due to a different ontogenetic origin, implying that cephalopod hooks evolved convergently more than once during their history and may not have originated from the same initial organ and thus, are not truly homologous. Indeed, hooks in belemnoids are thought to be homologous with cirri and trabeculae and not suckers^26^. Nonetheless, it is commonly accepted that the hook-like structures present on the brachial crowns of modern decabrachians and belemnoids performed a similar function i.e., prey grasping^23,26–28,41,42^. It seems therefore plausible that in ammonites as well, a far more distant relative of modern decapodiforms and belemnoids^43^, brachial crown hooks may have evolved convergently as well.

Although they have been subject to taphonomic processes, the spatial distribution of the hooks in the best preserved specimens of *Rhaeboceras halli* is consistent with an arrangement on the arm crown (i.e., the hooks are aligned in pairs, forming up to two distinct axes). With one exception (out of 50 reported occurrences), they are always preserved in the body chamber. Presumably, the arms would have retracted into the body chamber directly preceding (due to stress) or following the death of the animal^1^. In addition, in specimens that contain *in-situ* jaws, implying that the body was still inside the shell during fossilization, the hooks occur below the jaws, suggesting that they were derived from a ventral arm pair. Indeed, in some modern cephalopods, like *Sepia*, for example, the tentacles can retract into tentacular pockets slightly behind and below the jaws^44^.

The number of brachial crown hooks varies broadly among coleoids: 20 to 100 hooks on each of 10 arms in belemnoids^18,23,25^; 40 to 45 hooks per arm in *Ancistrocheirus lesueurii*^45,46^;15 to 25 hooks per arm in Enoplotheutidae^47,48^; 1 to 3 big hooks on the tentacular club in addition to smaller hooks along the arms I, II and III in the Gonatidae^35^; and 60 hooks or small suckers per tentacular club in the Onychoteuthidae^49^ (Fig. 4A,B). The total number of hooks per specimen in *Rhaeboceras halli* is also variable (40 to 168; Table S1). These values may represent underestimates since in some specimens of *R. halli,* not all the hooks were captured in the reconstruction. In other specimens, weathering may have destroyed the hooks that were originally present. Indeed, in the specimens with the most hooks (AMNH 66350 with 168 structures and AMNH 95795 with 122 structures), the majority of hooks are not exposed at the surface. Thus, the number of hooks in *R. halli* is comparable to the total number of tentacular hooks or arm hooks known in other cephalopods.

The grouping and imbrication of multiple hooks of the same morphotype and the paired relation between morphotypes in *Rhaeboceras halli* also support the hypothesis that they belong to the brachial crown, as it is reminiscent of the distribution of hooks on the arms and tentacles in modern decabrachians and belemnoids (closely aligned in pairs along the arms). The chirality between the morphotypes in *R. halli* highlighted by Kruta et al.^32^ is consistent with the chirality observed on the armature (i.e., the whole of the arm structures) of some specialised arms in modern decabrachians, such as in *Taonius pavo*, in which bicuspid hooks on the tentacles mirror each other^35^. Based on the attachment between the onychites and arms in belemnoids^23,25,34^ and the hooks and arms in modern decabrachians^23,35^, we can also assume that the hooks in *R.halli* were attached via their openings to soft tissue on the arms. Besides, given the multiple similarities between the opening in *R.halli* hooks and that of onychites (e.g., single sided slanted opening), the insertion of the hooks was more likely similar to that of the latter rather than to that of modern decabrachians. The persistent homology analyses support this hypothesis, with the hooks of the same morphotype describing up to two distinct axes in space and pointing outwards. They were thus probably aligned in pairs on the arms with the cusps pointing forward.

A surprising feature, however, is the broad variation in size and shape of the elements in *Rhaeboceras halli*. In extinct coleoids (e.g., Donovaniconida, Belemnitida, and Phragmoteuthida), some morphological variability has been reported^50^, yet no more than four morphotypes within a single individual have been identified. Besides, the morphological differentiation of these morphotypes appears to mainly be due to their curvature. In modern cephalopods, arm hooks are generally nearly uniform within a single individual and per arm. Structures of different morphology have occasionally been reported in arms that are modified for reproduction, i.e., hectocotyli^51^. A hectocotylus is a single modified arm for reproduction on which the suckers develop laterally in order to form a trench along the whole arm. This trench is then used to transfer the spermatophores into the mantle cavity of the female. After the process, some males are capable of self-amputation of their hectocotylus, which then remains in the female. This structure could therefore be found in the pallial cavity of females, as in argonauts where this feature is common^52^. However, given the size and number of hooks in *R. halli* (up to 168 in AMNH 66350), the likelihood that they belong to a single arm is low. In other extant decabrachians and belemnoids, giant hooks (Mega-onychites) found only as a single pair have been interpreted by several authors as mating structures used to hold the female during reproduction^12,53^.

Besides the hectocotylus, the only brachial crown structures that come close to the structures described here in terms of morphological variability are tentacular club structures. High morphological variability among tentacular structures is common in modern decabrachians^35^ where a differentiation can usually be observed between the structures on the carpus, manus, and dactylus and/or across the width of the tentacle, as in *Onychoteutis bankssi* (Fig. 4A,B). In extant Cranchiidae, structures show broad variation along the tentacular club, from little suckers with chitinous rings to enlarged bicuspid and even multicuspid teeth^37,38^ (Fig. 4D,E).

**Figure 6:**
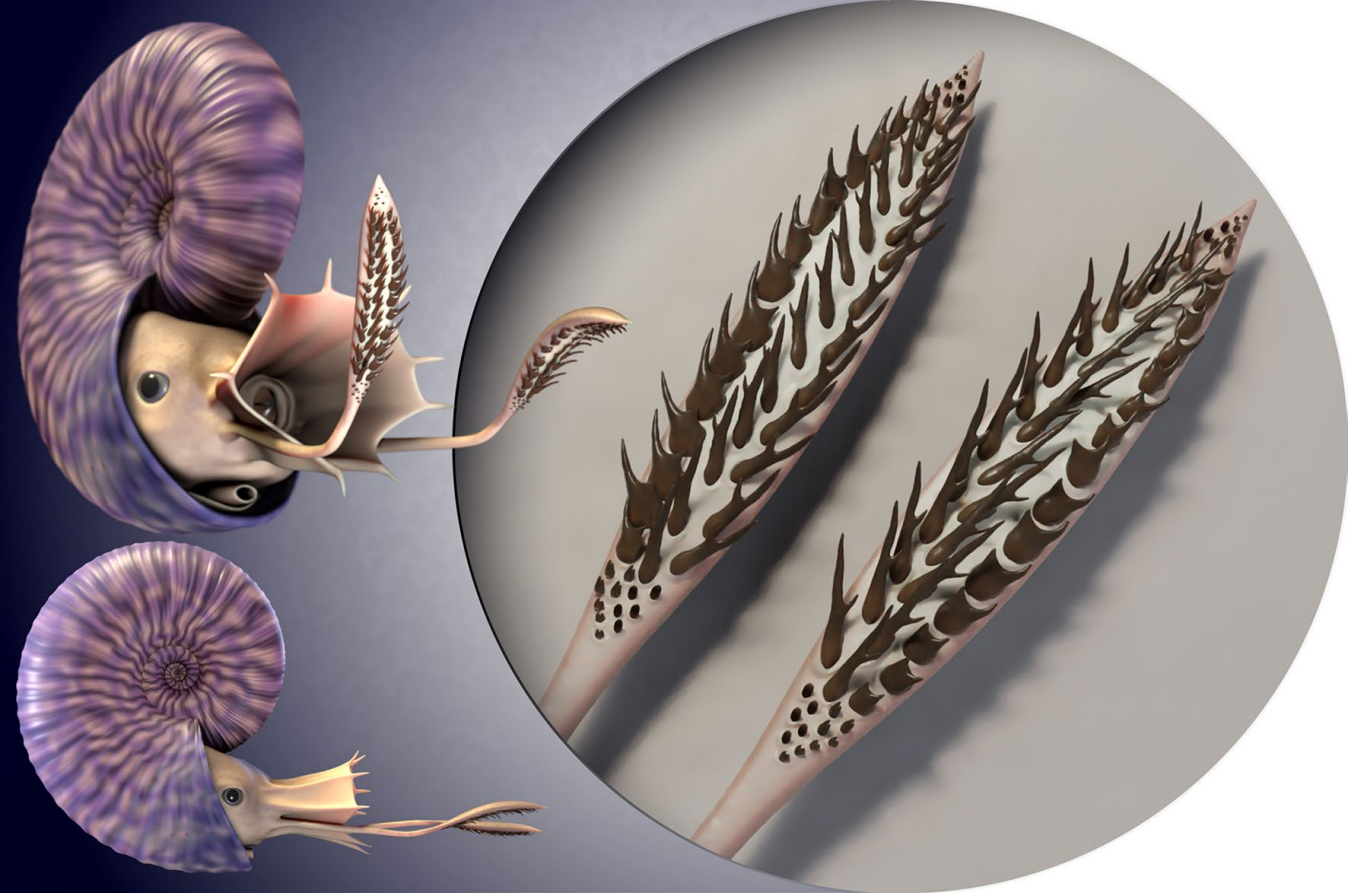
Hypothetical reconstruction of a *R. halli* tentacular club. The arrangement of the different morphotypes of hooks along the tentacular clubs was inferred based on the recurrent 3D patterns and morphotype associations identified in multiple specimens throughout this study, and comparisons with extant coleoid armatures. The size of the clubs is estimated, and falls within the range observed in modern coleoids. 3D reconstruction by A. Lethiers (CR2P).

The morphological variation among the different morphotypes of hooks in *Rhaeboceras halli*, their number per specimen, their size, their distribution within the body chamber, their arrangement, and the presence of up to two axes inferred from the persistent homology analysis supports the hypothesis of a pair of arms. More specifically, we envision tentacles with soft tissue inserted through the openings of the hooks to link them to the arm, rather than simple arm structures. This leads us to the following reconstruction of the hypothetical, original hook distribution in *R.halli* (Fig. 6).

## Conclusion

With their characteristically hollow, bicuspid shape and their single-sided, wide opening at the base, the structures in *Rhaeboceras halli* and *Hoploscaphites* are unique and very different from other known cephalopod structures, whether extant or extinct. Indeed, this is the first report of cephalopod-associated hook-like structures outside coleoids, restricting comparisons. Morphological variations are observable among the structures of different members of the Scaphitidae. Variability within individuals, which has yet to be formally described, has also been observed. Nonetheless, the nine major morphotypes described by Kruta et al.^32^ in *R. halli* have been confirmed. Study of the morphology of the hooks has already allowed the rejection of a radular origin^32^. Their precise nature, however, was up till now still uncertain. The study of their spatial distribution in *R. halli* provides new elements allowing us to clarify this matter. Recurrent patterns in the arrangement of the hooks, conspicuous in the best preserved specimens, have been highlighted. They are always located in the body chamber. Some morphotypes are associated with each other in pairs (G1&G4; G2&G5). These associated morphotypes define up to two distinct axes in space, with up to about 60 or 70 hooks per axis (maximum estimate); these topological features are all the more highlighted by the persistent homology analyses, emphasising the potential of topological data analyses applied to palaeontological material, especially given the growing popularity of topographical analysis. The morphology of the hooks varies along these axes, with the very small structures at the base, followed by the biggest structures. All these traits seem to indicate that the hooks are brachial crown structures. The comparison with other cephalopod structures further supports this hypothesis, as the previously described arrangement of hooks in *R. halli* is reminiscent of the distribution of structures known from decabrachian tentacle clubs. Given all this evidence, we come to the conclusion that the hook-like structures in *R*. *halli* do indeed represent arm structures and, most likely, tentacular club structures.

Armature hooks in belemnoids (i.e., onychites) and modern decabrachians are believed to be convergent acquisitions as they both serve as grasping devices related to feeding habits. It is plausible the structures in *Rhaeboceras halli* served the same function. One hypothesis is that *R.halli* developed some sort of ambush hunting strategy in which the hooks were used to clasp small prey despite being slow swimmers^54–56^, as perhaps suggested by the co-occurrence of hooks and fish remains preserved together in a single concretion (Fig. S5). Further work must however be conducted before being able to elucidate the exact function of these hooks, as grasping devices for mating remains, among others, a plausible hypothesis. Nonetheless, these structures are the very first ammonite brachial crown elements described, considerably improving our knowledge about the evolution of arms and their armature in cephalopods, and opening a whole new field of study in ammonite evolution and paleoecology.

## Material

The specimens of *Rhaeboceras halli* examined in this study are from the upper Campanian *Baculites jenseni* Zone^57^ of the Bearpaw Shale in northeast Montana. They are reposited at the American Museum of Natural History (AMNH), New York; the Black Hills Institute of Geological Research (BHI), Hill City, South Dakota; and the U.S. National Museum (USNM), Washington, D.C. We re-examined six specimens of *R*. *halli* previously studied by Kruta et al*.*^32^ AMNH 64405, 66350, 66351, 66433, 66434 and 66448. In addition, we examined 17 other *R. halli* specimens from the same site that also contained hooks. Of these, we concentrated on the six best preserved specimens and CT-scanned them: BHI 4818, AMNH 95795, 51333, 162970, 108408, and AMNH 160989. Only AMNH 95795 and AMNH 160989 provided satisfactory scan results. Three specimens (BHI 4818, AMNH 51333, and AMNH 162970) turned out to contain no hooks at all and one specimen (AMNH 108408) was too dense to provide exploitable tomographic data.

Six specimens of *Hoploscaphites* representing three species (*H. gilberti ?*, *H. nicolletii*, *H. comprimus*) were also examined (Table S2) and one was CT-scanned: *H. nicolletii* (AMNH 51333) from the upper Maastrichtian Fox Hills Formation, north-central South Dakota. It preserves part of the body chamber and the lower jaw is *in-situ*. The hooks are preserved in the matrix.

Scaphitid ammonites are sexually dimorphic^58^, with the larger morph (the macroconch) interpreted as the female, and the smaller morph (the microconch), interpreted as the male. We therefore interpret the studied specimens as macroconchs based on their large size and robust shape. Only two specimens of *Rhaeboceras halli* collected in the upper Campanian *Baculites jenseni* Zone of the Bearpaw Shale in northeast Montana have been interpreted as microconchs. Neither exhibits any hook-like structures. It should however be noted that some uncertainty persists regarding the recognition of males and females, as dimorphism has not yet been thoroughly studied in *Rhaeboceras*.

All of the specimens we examined (29 specimens) are internal moulds and retain part or all of the body chamber. The hooks occur inside the body chamber and although some of them are visible on the surface (Fig. 2A,B), most of them are still embedded in the matrix. We observed only one occurrence of hooks not in connection with an ammonite; the hooks are preserved in a small limestone concretion (15 cm in length) associated with a nearly complete fish skeleton (Fig. S5). The nature of this co-occurrence remains however unclear. A high proportion of the specimens with hooks also retain the jaws inside the body chamber (Table S2), which is interpreted as evidence of rapid burial after death.

To better interpret our results, we also investigated the morphology of modern and extinct cephalopods based on the literature and examination of actual specimens housed in the Yale Peabody Museum (YPM). We selected the species *Taonius pavo* (YPM 029245 and 037340) and *Onychoteuthis banksii* (YPM 17905, 17907, 17909 and 17911) for study due to the particular armature of their arms consisting of horny, unicuspid and bicuspid hooks.

## Methods

### Hook segmentation and identification

The cluster of hooks in each body chamber was revealed using µCT-scanning and propagation phase-contrast X-ray synchrotron microtomography (PPC-SR-µCT-ESRF proposal es-859). For more detail on data acquisition, refer to Kruta et al*.*^32^. The six newly studied specimens of *Rhaeboceras halli* were µCT-scanned at the AMNH using a GE PHOENIX v|tome|x s 240. The 3D segmentation was performed using VG studio Max 3.2 (Volume Graphics, Heidelberg, Germany). Most of the segmentation was performed using threshold tools.

The hooks are hollow and filled with the surrounding sedimentary matrix. They are composed of a thin wall of black material identified as the mineral brushite^32^. As a result, the density difference between the hooks and the surrounding matrix is high, facilitating their reconstruction with threshold tools. However, when the hooks were partly exposed on the surfaces of specimens, segmentation was performed manually.

After further examination of the different hook morphologies and the morphological disparity of the hooks studied by Kruta et al.^32^, and taking into account the morphotypes that had previously been defined, each newly reconstructed hook was assigned to a morphotype.

### Study of spatial distribution

In order to better comprehend the distribution of the hooks in the body chamber and their relations between each other, the same landmarks used in Kruta et al.^32^ were positioned on the reconstructed hooks of the newly CT scanned specimens. The coordinates of these landmarks were then exported along with the coordinates of the landmarks used in Kruta et al.^32^ and analysed using R software (R Core Team 2016).

Multiple aspects of the spatial distribution of the hooks were examined based on observations and the centroid position of each hook derived from the landmarks: (i) The overall position of the hooks in the body chamber was studied through examination of 8 CT-scanned specimens. In addition, 18 specimens where also examined on the outside. (ii) The reconstructions of the hooks obtained from the CT-scan data using VGL 3.2 Volume Graphics (Heidelberg, Germany), a 3D data visualisation software, were used to validate the morphotypes described in Kruta et al.^32^, and describe the general arrangement of the structures in space within the body chamber. (iii) Using statistical analyses, the position of each hook was studied in relation with other hooks of the same morphotype, as well as with hooks of different morphotypes.

### Statistical analyses

The approach used here to describe the relationships between hooks is based on their centroids. The centroid of each hook provides the best estimate of the position of the hook inside the body chamber. We used the centroid of the four landmarks of the opening, as we assume it corresponds to the position of the soft tissue attachment. In order to identify the geometrical arrangement of the hooks, we used a method derived from persistent homology, which is a new topological data analysing method that has only recently been applied in a few fields such as neurology^59^, molecular chemistry^60–62^, and material sciences^63^ but never, as far as we know, in paleontology. This method consists in establishing links between points in space based on their proximity in order to highlight possible pathways between them (for more detail see Supplementary Material section “presentation of persistent homology”). To do so we used functions from the R package TDA^64^.

To investigate the spatial relationships among morphotypes, we examined the distances between the hooks. In each specimen, and for each hook, we first searched for its closest neighbour among all the hooks, including those of the same morphotype and then, only among hooks of a different morphotype. Our hypotheses are that (i) if hooks are clustered per morphotype, the closest neighbour to any hook of morphotype *m*_*i*_ should most of the time be a hook of that same morphotype *m*_*i*_, (ii) that if any morphotypes *m*_*i*_ and *m*_*j*_ are related, then the closest neighbour to any hook of morphotype *m*_*i*_ and from a different morphotype than *m*_*i*_ should most often be a hook of morphotype *m*_*j*_ rather than of any other morphotype and *vice versa*. To test these hypotheses, we computed the distances between the centroids of all hooks in each specimen. Then, for all hooks of each morphotype *m*_*i*_, (i) we first counted the number of times the closest hook belonged to the same morphotype *m*_*i*_, (ii) and then counted the number of times the closest hook belonged to each of the other morphotypes *m*_*j*_ after excluding the relation *m*_*i*_-*m*_*i*_. Given the results in (i) (i.e., the structures are clustered by morphotype), in order to avoid biasing the analyse in (ii) we excluded the outlier hooks of each morphotype based on their distance to the other hooks of the same morphotype using the 1.5xIQR rule. In other words, the hooks that are far apart from the cluster formed by the other hooks of the same morphotype were excluded. We compared these counts to the null hypothesis value:

- For (i) $\mathop \sum \limits_{specimens} Em_{i - i}$, where $Em_{i - i}$ is the estimated number of hooks of morphotype $m_{i}$ that have as closest neighbour a hook of the same morphotype $m_{i}$ ,assuming the hooks are randomly distributed. For each specimen, $Em_{i - i} = { }(Nm_{i} - 1)/\left( {N_{tot} - 1} \right) \times Nm_{i}$ with $Nm_{i}$ corresponding to the number of hooks of morphotype *m*_*i*_ and $N_{tot}$ corresponding to the number of hooks in the specimen.

- For (ii) $\mathop \sum \limits_{specimens} Em_{i - j}$, where $Em_{i - j}$ is the estimated number of hooks of morphotype $m_{i}$ that have as closest neighbour a hook of morphotype $m_{j}$ after excluding outliers and the other hooks of morphotype $m_{i}$_,_ assuming the hooks are randomly distributed. For each specimen, $Em_{i - j} = { }N^{\prime}m_{j} /\left( {N^{\prime}_{tot} - N^{\prime}m_{i} } \right) \times N^{\prime}m_{i}$ with $N^{\prime}m_{i}$ corresponding to the number of hooks of morphotype $m_{i}$ after excluding outliers*,* $N^{\prime}m_{j}$ corresponding to the number of hooks of morphotype $m_{j}$ after excluding outliers, and $N^{\prime}_{tot}$ corresponding to the number of hooks in the specimen after excluding outliers.

Finally, the ratio of the observed values to the null hypothesis values indicates the deviation from a random distribution scenario. The higher these ratios (expressed as a percentage) are (i) the better the hooks of the same morphotype are clustered and (ii) the stronger the relationship between morphotype $m_{i}$ and $m_{j}$ is. To make the procedure as clear as possible, an example for each hypothesis testing is provided in Supplementary Material.

## Supplementary Information

Supplementary Video 1.

Supplementary Information 1.

Supplementary Video 2.

Supplementary Information 2.

Supplementary Video 3.

Supplementary Information 3.

Supplementary Video 4.

Supplementary Information 4.

Supplementary Video 5.

Supplementary Information 5.

Supplementary Video 6.

Supplementary Information 6.

Supplementary Video 7.

Supplementary Information 7.

Supplementary Information 8.
