# Supplementary figures and images for: New evidence from exceptionally “well-preserved” specimens sheds light on the structure of the ammonite brachial crown
Source: Sci Rep. 2021 Jun 4;11:11862. doi: 10.1038/s41598-021-89998-4 (PMC8178333; doi:10.1038/s41598-021-89998-4)

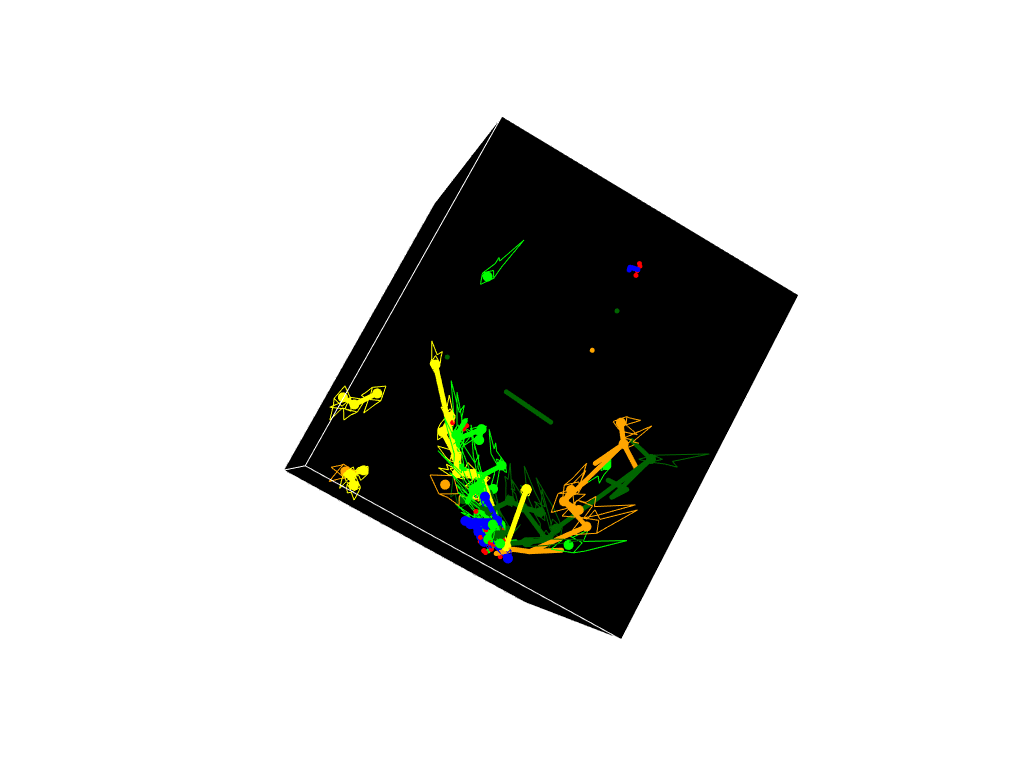

Supplement: Supplementary file 1 — Supplementary Video 1. [file 41598_2021_89998_MOESM1_ESM.gif]

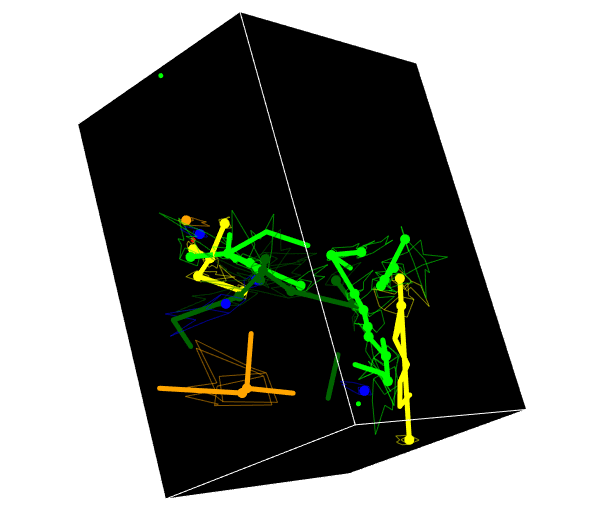

Supplement: Supplementary file 3 — Supplementary Video 2. [file 41598_2021_89998_MOESM3_ESM.gif]

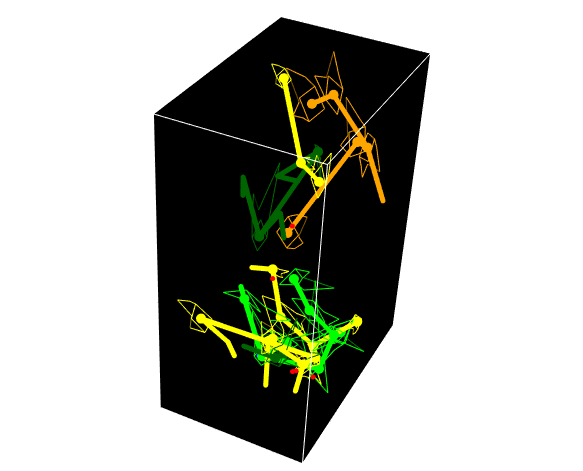

Supplement: Supplementary file 5 — Supplementary Video 3. [file 41598_2021_89998_MOESM5_ESM.gif]

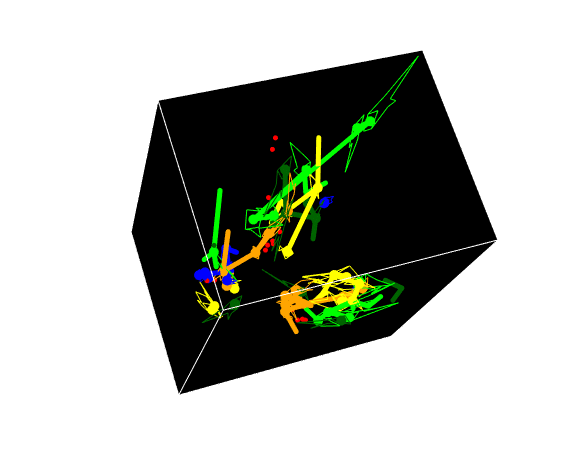

Supplement: Supplementary file 7 — Supplementary Video 4. [file 41598_2021_89998_MOESM7_ESM.gif]

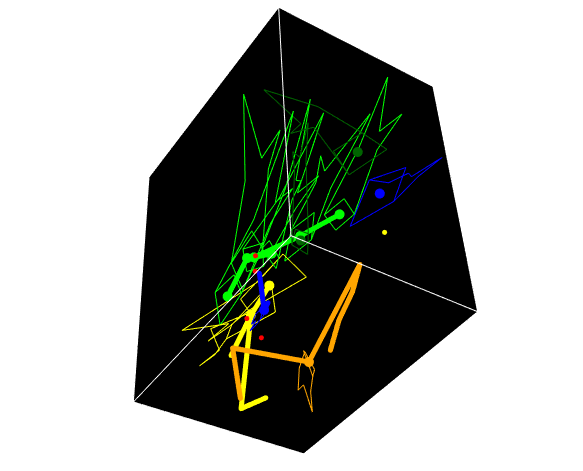

Supplement: Supplementary file 9 — Supplementary Video 5. [file 41598_2021_89998_MOESM9_ESM.gif]

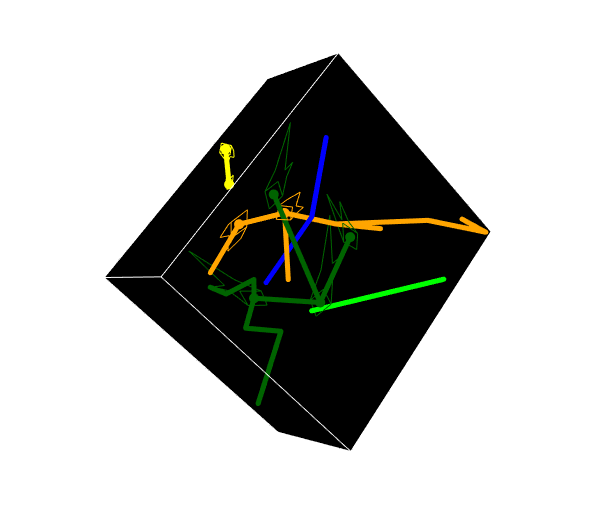

Supplement: Supplementary file 11 — Supplementary Video 6. [file 41598_2021_89998_MOESM11_ESM.gif]

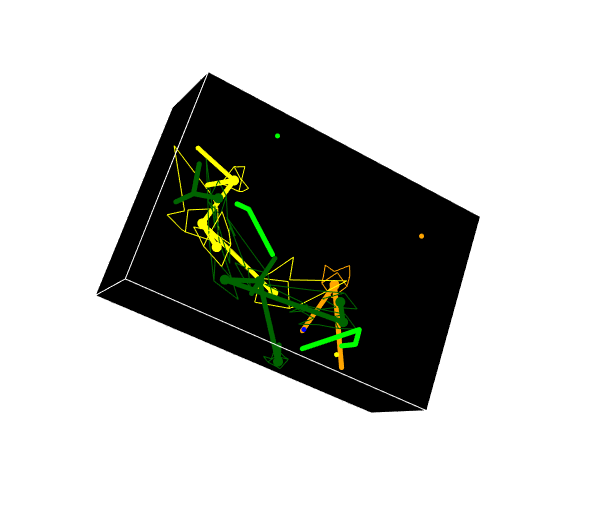

Supplement: Supplementary file 13 — Supplementary Video 7. [file 41598_2021_89998_MOESM13_ESM.gif]
