# Supplementary material for: New evidence from exceptionally “well-preserved” specimens sheds light on the structure of the ammonite brachial crown
Source: Sci Rep. 2021 Jun 4;11:11862. doi: 10.1038/s41598-021-89998-4 (PMC8178333; doi:10.1038/s41598-021-89998-4)
Supplement: Supplementary file 2 — Supplementary Information 1. [file 41598_2021_89998_MOESM2_ESM.html]

 
RGL model


You must enable Javascript to view this page properly.

  
Drag mouse to rotate model. Use mouse wheel or middle button
to zoom it.

---

  
Object written from rgl 0.100.50 by writeWebGL.
