# Supplementary material for: New evidence from exceptionally “well-preserved” specimens sheds light on the structure of the ammonite brachial crown
Source: Sci Rep. 2021 Jun 4;11:11862. doi: 10.1038/s41598-021-89998-4 (PMC8178333; doi:10.1038/s41598-021-89998-4)
Supplement: Supplementary file 15 — Supplementary Information 8. [file 41598_2021_89998_MOESM15_ESM.docx]

Supplementary Material

for

**New evidence from exceptionally “well-preserved” specimens shed light on the structure of the ammonite brachial crown**

C. P. A. Smith, N. H. Landman, J. Bardin, I. Kruta

[Brief presentation of persistent homology 2](#_Toc70673338)

[Example of morphotype clustering testing (i): 4](#_Toc70673339)

[Example of morphotype association testing (ii): 4](#_Toc70673340)

[Table S1. 6](#_Toc70673341)

[Table S2. 7](#_Toc70673342)

[Table S3. 8](#_Toc70673343)

[Table S4. 10](#_Toc70673344)

[Figure S2 11](#_Toc70673345)

[Figure S3 12](#_Toc70673346)

[Figure S4 13](#_Toc70673347)

[Figure S5. 14](#_Toc70673348)

[Figure caption for complementary animated and interactive plots: 15](#_Toc70673349)

# Brief presentation of persistent homology

Persistent homology is a method used in Topological Data Analyses (TDA) to extract topological features from multi-dimensional data^1^. The idea emerged in 1990 with the work of Patrizio Frosini^2^ and was completed in 1999 by Vanessa Robins^3^ who defined the idea of persistence in shape (for more details of the state of art of persistent homology, refer to Edelsbrunner & Harer^4^ and Carlsson^5^). Since then, the method has developed rapidly. However, it is essentially used on big data sets in molecular chemistry, material science and the medical field, especially in neurology, but remains poorly known elsewhere.

The principal of persistent homology is to compute, and follow the evolution of topological features through a gradually increasing filtration (threshold). The topological features are constructed by combining the components (the data as points/vertices) that connect under that filtration (**Fig.S1**). The components then fuse to form a more complex component (simplicial complex). The topological features thus appear (birth), disappear (die), fuse, and become more complex throughout the increasing filtration (**Fig.S1**). The longer the topological feature lasts (represented in bar code graphs), the more it is representative of the data. The topological features are sorted by their Betti number (β) which refers to the number of holes of each dimension. In other words, β_0_ indicates the number of components, with the simplest ones being simplices and/or pathways; β_1_ corresponds to structures presenting a circularity (1D holes); and β_2_ corresponds to structures with voids or cavities (2D holes).

Persistent homology is usually applied on big data sets to extract the main topological features (long lived features) and eliminate the noise (short lived features). In this study however, the goal is to extract one or several topological feature(s) that would correspond to the structure on which hooks are inserted. Thus, we started by focusing on only the two first betti number topological features in search of a circular pattern. None appeared. We then focused simply on the β_0_ topological features to extract the most representative pathway that would be indicative of an alignment and thus, axis. To do so, independently for each specimen and for each morphotype, we carried out a persistent homology analysis using the centroid position of the 4 landmarks positioned on the opening of the hooks where the soft tissue was probably attached.


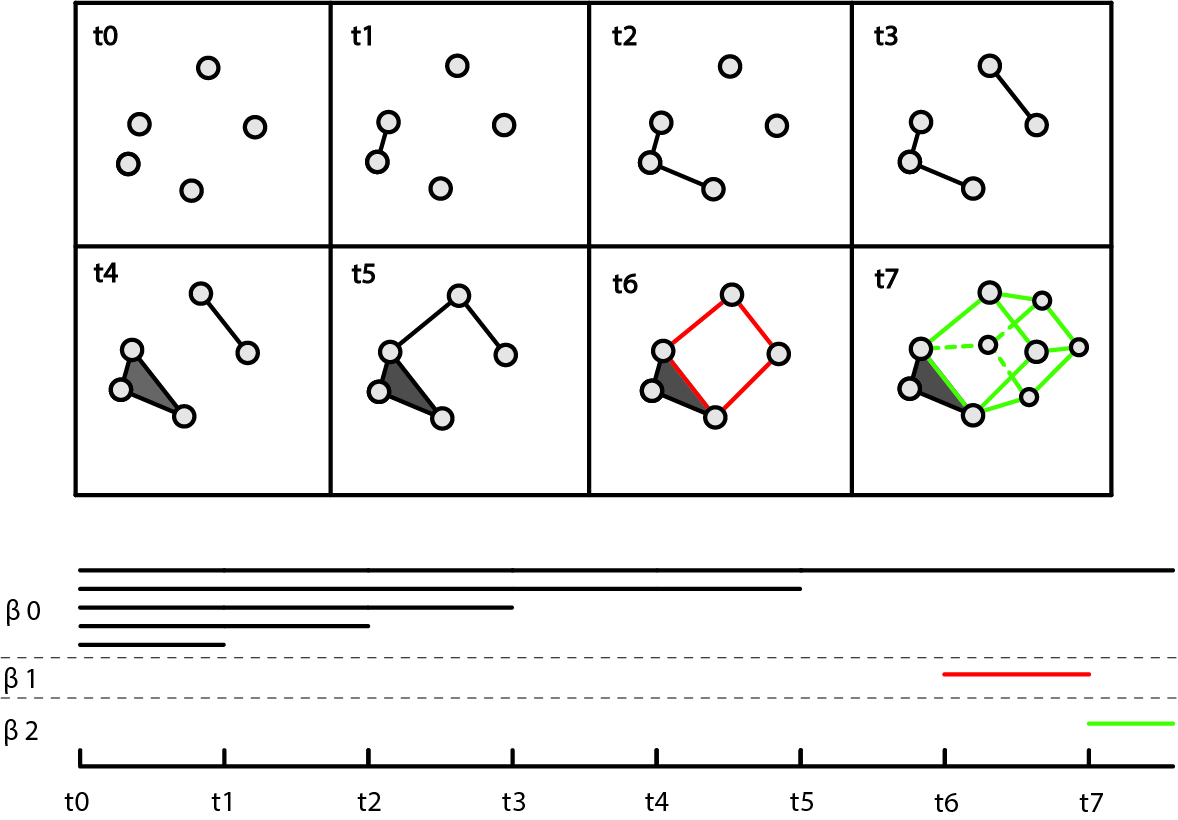


t0: 5 structures ( 5 x 0-simplex/vertex)

t1: 4 structures (3 x 0-simplex/vertex & 1 x 1-simplex/edge)

t2: 3 structures (2 x 0-simplex/vertex & 1 x 1-simplex/edge)

t3: 2 structures (2 x 1-simplex/edge)

t4: 2 structures (2 x 1-simplex/edge & 1 x 2-simplex/triangle)

t5: 1 structures (1 x 1-simplicial complex)

t6: 1 structures (1 x simplicial complex with hole)

t7: 1 structures (1 x simplicial complex with void)

**A**

**B**

**C**

**Figure S1**. Example of persistent homology filtration. **A** **–** Evolution of the connections established by the persistent homology throughout a gradually increasing filtration. **B** **–** Barcode graphic corresponding to the filtration presented in **A**. **C –** Detail of the constructed structures at each step of the filtration presented in **A**.

1. Otter, N., Porter, M. A., Tillmann, U., Grindrod, P. & Harrington, H. A. A roadmap for the computation of persistent homology. *EPJ Data Sci.* **6**, 17 (2017).

2. Frosini, P. A distance for similarity classes of submanifolds of a Euclidean space. *Bull. Austral. Math. Soc.* **42**, 407–415 (1990).

3. Robins, V. Towards computing homology from finite approximations. in *Topology proceedings* vol. 24 503–532 (1999).

4. Edelsbrunner, H. & Harer, J. Persistent homology—a survey. in *Contemporary Mathematics* (eds. Goodman, J. E., Pach, J. & Pollack, R.) vol. 453 257–282 (American Mathematical Society, 2008).

5. Carlsson, G. Persistent Homology and Applied Homotopy Theory. *arXiv:2004.00738 [math]* (2020).

# Example of morphotype clustering testing (i):

In this example, we want to verify and quantify if the hooks of morphotype 1 (M1) are clustered; in total there are 117 hooks belonging to M1. By computing the distances between all hooks and selecting the closest to each M1 hook in each specimen, we reveal that 59 M1 hooks have another M1 hook as closest neighbour. The null hypothesis which is the total expected number of M1 hooks having as closest neighbour another M1 hook if the hooks were randomly distributed is:

${Em}_{M1-M1 tot}$= $\sum_{specimens} {Em}_{M1-M1}$

${Em}_{M1-M1 tot}$= ${Em}_{M1-M1}$_AMNH66350_ + ${Em}_{M1-M1}$_AMNH95795_ + ${Em}_{M1-M1}$_AMNH66448_ + ${Em}_{M1-M1}$_AMNH6633_ + ${Em}_{M1-M1}$_AMNH66434_ + ${Em}_{M1-M1}$_AMNH64405_ + ${Em}_{M1-M1}$_AMNH66351_

${Em}_{M1-M1 tot}$=$\left( \frac{39-1}{147-1}\times39 \right)+\left( \frac{24-1}{102-1}\times24 \right)+\left( \frac{6-1}{32-1}\times6 \right)+\left( \frac{28-1}{65-1}\times28 \right)+\left( \frac{10-1}{51-1}\times10 \right)+\left( \frac{8-1}{32-1}\times8 \right)+\left( \frac{2-1}{26-1}\times2 \right)$

${Em}_{M1-M1 tot}$=10.151 + 5.465 + 0.968 + 11.813 + 1.800 + 1.806 + 0.080 = **32.083**

${Em}_{M1-M1 tot}$ corresponds to the estimated number of hooks of morphotype M_1_ that have as closest neighbour a hook of the same morphotype M_1_, all reconstructed specimens included. Table 1A shows the ratios of the observed values to the expected values. For M1, the ratio observed/expected = 59/32.083 = 1.84; as in percentages, this means that our observation on M1 hooks as closest neighbours to other M1 hooks are 184% that of what would be expected with a random distribution of hooks in space. Such results confirm the M1 hooks are not randomly distributed, but are indeed clustered.

# Example of morphotype association testing (ii):

In this example, we want to verify if there is a relation between morphotype 1 (M1) and morphotype 4 (M4). By computing the distances between all hooks after excluding the outliers, and excluding the possibility of M1 hooks being the closest neighbours to other M1 hooks, we reveal that 44 M1 hooks have as closest neighbour a M4 hook. The null hypothesis which is the total expected number of M1 hooks having as closest neighbour a M4 hook after excluding the outliers, and excluding the possibility of M1 hooks being the closest neighbours to other M1 hooks if the hooks were randomly distributed is:

${Em}_{M1-M4 tot}$= $\sum_{specimens} {Em}_{M1-M4}$

${Em}_{M1-M4 tot}$= ${Em}_{M1-M4}$_AMNH66350_ + ${Em}_{M1-M4}$_AMNH95795_ + ${Em}_{M1-M4}$_AMNH66448_ + ${Em}_{M1-M4}$_AMNH6633_ + ${Em}_{M1-M4}$_AMNH66434_ + ${Em}_{M1-M4}$_AMNH64405_ + ${Em}_{M1-M4}$_AMNH66351_

${Em}_{M1-M4 tot}$= $\left( \frac{18}{115-27}\times27 \right) + \left( \frac{19}{97-24}\times24 \right) + \left( \frac{5}{27-5}\times5 \right) + \left( \frac{14}{62-27}\times27 \right) + \left( \frac{12}{43-10}\times10 \right) + \left( \frac{5}{27-7}\times7 \right) + \left( \frac{0}{22-0}\times0 \right)$

= 5.523 + 6.247 + 1.136 + 10.8 + 3.636 + 1.644 + 0 = **28.986**

${Em}_{M1-M4 tot}$corresponds to the estimated number of hooks of morphotype M_1_ that have as closest neighbour a hook of morphotype M_4,_ , all reconstructed specimens included. Table 1B shows the ratios of the observed values to the expected values. For the relation M1-M4, the observed/expected = 44/28.986 = 1.52; as in percentages, this means that our observation on M4 hooks as closest neighbours to M1 hooks are 152% that of what would be expected with a random distribution of hooks in space. Besides, M1 hooks are also more often the closest neighbour to hooks of morphotype M4 than expected if the hooks had been randomly distributed. Therefore, it seems indeed that morphotype 1 and morphotype 4 are associated with each other.

|  | AMNH  95795 | AMNH  64405 | AMNH  66448 | AMNH  66350 | AMNH  66351 | AMNH  66433 | AMNH  66434 |
| --- | --- | --- | --- | --- | --- | --- | --- |
| *Hooks used for landmarks* | | | | | | | |
| G1 | 11 |  | 6 | 27 |  | 18 | 9 |
| G2 | 14 | 1 | 1 | 8 | 2 | 3 | 5 |
| G3 | 7 |  | 2 | 11 |  | 4 |  |
| G4 | 11 | 4 | 2 | 17 | 2 | 8 | 12 |
| G5 | 8 | 6 | 2 | 17 | 4 | 8 | 4 |
| Total | **51** | **11** | **12** | **80** | **8** | **41** | **30** |
| *Identifiable hooks, but too incomplete or small to be used for landmarks* | | | | | | | |
| Probably G1 | 13 | 8 |  | 12 | 2 | 10 | 1 |
| Probably G2 | 3 | 3 | 6 | 7 | 7 | 3 | 1 |
| Probably G3 | 3 | 1 | 2 | 5 | 3 |  | 1 |
| Probably G4 | 8 | 3 | 4 | 1 |  | 6 | 5 |
| Probably G5 | 6 | 6 |  | 9 | 6 | 4 | 8 |
| Unicuspid |  |  |  | 3 |  |  |  |
| Tricuspid | 4 |  | 3 |  |  |  |  |
| Very small | 14 |  | 4 | 30 |  | 1 | 5 |
| Rounded |  |  |  | 1 |  |  |  |
| *Unidentifiable Hooks* | | | | | | | |
|  | 20 | 16 | 15 | 20 | 14 | 10 |  |
| *Total number of hooks per specimen* | | | | | | | |
|  | 122 | 48 | 47 | 168 | 40 | 75 | 51 |

Table S1. Summary of the hook-like structures discovered in each specimen. Hooks were attributed to their respective morphotype following Kruta et al., 2020. “Incomplete” and very small hooks could not be used for landmarks but we were able to assign most of them to a morphotype. However, some hooks too poorly preserved remain unidentified.

| **Specimen** | **Species** | **Lateral location** | **Antero-posterior location** | **Lower Jaw** |
| --- | --- | --- | --- | --- |
|  |  |  |  |  |
| **AMNH45972** | *R. halli* | - | - | Absent |
| **AMNH63530 - 1 hook** | *H. gilberti* ? | - | - | - |
| **AMNH66224** | *R. halli* | Right | Middle | Present, near aperture, hooks far behind |
| **AMNH66348** | *R. halli* | Venter | - | Absent |
| **AMNH66349** | *R. halli* | - | - | Absent |
| **AMNH66350*** | *R. halli* | Left | Slightly posterior | Absent |
| **AMNH66351*** | *R. halli* | Right flank | Middle | Absent |
| **AMNH66405*** | *R. halli* | Left flank | Middle | Absent |
| **AMNH66433*** | *R. halli* | Left flank | Slightly anterior | Present at the aperture |
| **AMNH66434*** | *R. halli* | Left flank and venter | Slightly anterior | Absent |
| **AMNH66448*** | *R. halli* | Left flank | Posterior | Absent |
| **AMNH66462** | *R. halli* | Left | Middle | Absent |
| **AMNH84331** | *R. halli* | Venter | Slightly posterior | Present; hooks behind |
| **AMNH95795*** | *R. halli* | Venter (2 clusters: one slightly on the right flank and the other one on the venter) | Middle | Absent |
| **AMNH95806** | *R. halli* | Venter | Posterior | Absent |
| **AMNH95808** | *R. halli* | Venter | Middle | Present; hooks behind |
| **AMNH95815** | *R. halli* | Right flank | - | Absent |
| **AMNH95826** | *R. halli* | - | - | Present |
| **AMNH95827** | *R. halli* | - | - | Absent |
| **AMNH95828** | *R. halli* | - | - | Absent |
| **AMNH108489** | *R. halli* | Left flank | - | Present; hooks behind |
| **AMNH108490** | *R. halli* | Left | Middle | Absent |
| **AMNH160988** | *R. halli* | Left flank | Middle | Present; hooks behind |
| **AMNH160989*** | *R. halli* | Slightly right flank | Posterior | Absent |
| **BHI 2156** | *R. halli* | - | - | Present; hooks below |
| **YPM32337 - 1 hook** | *H. nicolletii* | - | Near apt. | Absent |
| **YPM32354 - 1 hook** | *H. nicolletii* | - | Near apt. | Absent |
| **AMNH51333*** | *H. nicolletii* | Venter | Middle | Present, hook below |
| **YPM23239 - 3 hooks** | *H. nicolletii* | - | Near apt. | Absent |

Table S2. Summary of the studied specimens. For each specimen, the location of the hook cluster within the body chamber is specified. In specimens where the lower jaw has been reported, the relative position of the hook cluster to the jaw is also indicated. The asterisk indicates CT-scanned specimens.

Table S3. Detail per specimens of the nearest neighbour to each hook based on the centroid position of the hooks, taking all hooks into consideration, including those of the same morphotype. The colour scale is based on the number in parentheses, which represents the ratio observations / expected if randomly distributed. It does not apply to the unicuspid and tricuspid morphotypes due to too few representatives.

|  | **M1** | **M2** | **M3** | **M4** | **M5** | **M6** | **Unicuspid** | **Tricuspid** |
| --- | --- | --- | --- | --- | --- | --- | --- | --- |
| **AMHN66350** |  |  |  |  |  |  |  |  |
| **M1** | 19 (187 %) | 2 (50 %) | 1 (23 %) | 6 (125 %) | 3 (43 %) | 8 (100 %) | 0 |  |
| **M2** | 2 (50 %) | 6 (417 %) | 0 | 2 (108 %) | 3 (112 %) | 2 (65 %) | 0 |  |
| **M3** | 2 (47 %) | 0 | 6 (365 %) | 0 | 1 (35 %) | 6 (183 %) | 1 (304 %) |  |
| **M4** | 6 (125 %) | 1 (54 %) | 0 | 6 (286 %) | 2 (62 %) | 3 (81 %) | 0 |  |
| **M5** | 6 (86 %) | 2 (75 %) | 0 | 1 (31 %) | 15 (337 %) | 2 (37 %) | 0 |  |
| **M6** | 5 (62 %) | 1 (32 %) | 5 (152 %) | 2 (54 %) | 2 (37 %) | 14 (235 %) | 1 (162 %) |  |
| **Unicuspid** | 0 | 1 (324 %) | 1 (304 %) | 0 | 0 | 1 (162 %) | 0 |  |
| **AMNH95795** |  |  |  |  |  |  |  |  |
| **M1** | 13 (238 %) | 3 (74 %) | 0 | 3 (66 %) | 5 (150 %) | 0 |  | 0 |
| **M2** | 5 (124 %) | 3 (111 %) | 3 (178 %) | 1 (31 %) | 3 (127 %) | 2 (85 %) |  | 0 |
| **M3** | 2 (84 %) | 3 (178 %) | 3 (337 %) | 0 | 0 | 2 (144 %) |  | 0 |
| **M4** | 6 (133 %) | 4 (125 %) | 0 | 7 (207 %) | 2 (76 %) | 0 |  | 0 |
| **M5** | 2 (60 %) | 3 (127 %) | 2 (144 %) | 2 (76 %) | 5 (277 %) | 0 |  | 0 |
| **M6** | 0 | 0 | 3 (216 %) | 0 | 0 | 10 (555 %) |  | 1 (180 %) |
| **Tricuspid** | 0 | 0 | 1 (253 %) | 0 | 1 (180 %) | 0 |  | 2 (1683 %) |
| **AMHN66448** |  |  |  |  |  |  |  |  |
| **M1** | 2 (207 %) | 0 | 1 (129 %) | 0 | 1 (258 %) | 2 (258 %) |  | 0 |
| **M2** | 0 | 5 (369 %) | 0 | 1 (74 %) | 0 | 0 |  | 1 (148 %) |
| **M3** | 1 (129 %) | 0 | 2 (517 %) | 0 | 0 | 1 (194 %) |  | 0 |
| **M4** | 0 | 1 (74 %) | 0 | 5 (517 %) | 0 | 0 |  | 0 |
| **M5** | 2 (517 %) | 0 | 0 | 0 | 0 | 0 |  | 0 |
| **M6** | 1 (129 %) | 0 | 1 (194 %) | 0 | 0 | 0 |  | 2 (517 %) |
| **Tricuspid** | 0 | 1 (148 %) | 0 | 0 | 0 | 2 (517 %) |  | 0 |
| **AMNH66433** |  |  |  |  |  |  |  |  |
| **M1** | 17 (144 %) | 0 | 1 (57 %) | 7 (114 %) | 3 (57 %) | 0 |  |  |
| **M2** | 0 | 2 (427 %) | 2 (533 %) | 0 | 2 (178 %) | 0 |  |  |
| **M3** | 1 (57 %) | 0 | 0 | 0 | 2 (267 %) | 1 (1600 %) |  |  |
| **M4** | 6 (98 %) | 0 | 0 | 6 (211 %) | 1 (38 %) | 1 (457 %) |  |  |
| **M5** | 4 (76 %) | 1 (89 %) | 2 (267 %) | 1 (38 %) | 4 (194 %) | 0 |  |  |
| **M6** | 0 | 0 | 0 | 1 (457 %) | 0 | 0 |  |  |
| **AMNH66434** |  |  |  |  |  |  |  |  |
| **M1** | 5 (278 %) | 0 | 1 (500 %) | 2 (59 %) | 1 (42 %) | 1 (100 %) |  |  |
| **M2** | 0 | 5 (833 %) | 0 | 0 | 0 | 1 (167 %) |  |  |
| **M3** | 1 (500 %) | 0 | 0 | 0 | 0 | 0 |  |  |
| **M4** | 3 (88 %) | 1 (49 %) | 0 | 6 (110 %) | 2 (49 %) | 5 (294 %) |  |  |
| **M5** | 2 (83 %) | 1 (69 %) | 1 (417 %) | 1 (25 %) | 6 (227 %) | 1 (83 %) |  |  |
| **M6** | 0 | 1 (167 %) | 0 | 2 (118 %) | 0 | 2 (500 %) |  |  |
| **AMNH64405** |  |  |  |  |  |  |  |  |
| **M1** | 3 (166,07%) | 1 (97%) | 0 | 2 (111%) | 2 (65%) |  |  |  |
| **M2** | 0 | 1 (258%) | 1 (775%) | 1 (111%) | 1 (65%) |  |  |  |
| **M3** | 0 | 1 (775%) | 0 | 0 | 0 |  |  |  |
| **M4** | 1 (55%) | 0 | 0 | 1 (74%) | 5 (185%) |  |  |  |
| **M5** | 2 (65%) | 1 (65%) | 0 | 3 (111%) | 6 (141%) |  |  |  |

**< 120 % <**

**< 150 % <**

| **AMNH66351** |  |  |  |  |  |  |  |  |
| --- | --- | --- | --- | --- | --- | --- | --- | --- |
| **M1** | 0 | 1 (139 %) | 0 | 0 | 1 (125 %) |  |  |  |
| **M2** | 0 | 3 (104 %) | 1 (93 %) | 1 (139 %) | 4 (111 %) |  |  |  |
| **M3** | 0 | 1 (93 %) | 0 | 0 | 2 (167 %) |  |  |  |
| **M4** | 0 | 0 | 0 | 2 (2500 %) | 0 |  |  |  |
| **M5** | 1 (125 %) | 3 (83 %) | 2 (167 %) | 0 | 4 (111 %) |  |  |  |
|  |  |  |  |  |  |  |  |  |

Table S4. Detail per specimens of the nearest neighbour to each hook based on the centroid position of the hooks, only taking into consideration hooks of a different morphotype and after excluding outliers. The colour scale is based on the number in parentheses, which represents the ratio observations / expected if randomly distributed. It does not apply to the unicuspid and tricuspid morphotypes due to too few representatives.

**< 120 % <**

**< 150 % <**

|  | | **M1** | | **M2** | **M3** | | **M4** | | **M5** | | **M6** | | **Unicuspid** | | |  |
| --- | --- | --- | --- | --- | --- | --- | --- | --- | --- | --- | --- | --- | --- | --- | --- | --- |
| **AMNH66350** |  | |  | | |  | |  | |  | |  | |  |  | |
| **M1** | 0 | | 0 | | | 2 (65%) | | 9 (163%) | | 9 (128%) | | 6 (93%) | | 1 (109%) |  | |
| **M2** | 0 | | 0 | | | 0 | | 1 (44%) | | 9 (307%) | | 2 (75%) | | 1 (262%) |  | |
| **M3** | 2 (78%) | | 0 | | | 0 | | 0 | | 0 | | 5 (250%) | | 3 (1050%) |  | |
| **M4** | 4 (80%) | | 11 (456%) | | | 0 | | 0 | | 2 (47%) | | 1 (26%) | | 0 |  | |
| **M5** | 9 (133%) | | 10 (308%) | | | 1 (40%) | | 2 (44%) | | 0 | | 1 (19%) | | 0 |  | |
| **M6** | 7 (116%) | | 1 (34%) | | | 8 (358%) | | 1 (25%) | | 1 (19%) | | 0 | | 3 (448%) |  | |
| **Unicuspid** | 0 | | 1 (287%) | | | 1 (373%) | | 0 | | 0 | | 1 (178%) | | 0 |  | |
| **AMNH95795** |  | |  | | |  | |  | |  | |  | |  |  | |
| **M1** | 0 | | 3 (54%) | | | 1 (51%) | | 11 (176%) | | 6 (130%) | | 2 (43%) | |  | 1 (101%) | |
| **M2** | 7 (137%) | | 0 | | | 1 (78%) | | 1 (25%) | | 5 (168%) | | 3 (101%) | |  | 0 | |
| **M3** | 1 (63%) | | 2 (178%) | | | 0 | | 0 | | 0 | | 3 (325%) | |  | 0 | |
| **M4** | 7 (120%) | | 8 (193%) | | | 0 | | 0 | | 4 (117%) | | 0 | |  | 0 | |
| **M5** | 5 (124%) | | 3 (105%) | | | 1 (99%) | | 5 (156%) | | 0 | | 0 | |  | 0 | |
| **M6** | 3 (74%) | | 2 (70%) | | | 2 (198%) | | 2 (62%) | | 5 (212%) | | 0 | |  | 0 | |
| **Tricuspid** | 0 | | 1 (184%) | | | 1 (522%) | | 0 | | 0 | | 1 (224%) | |  | 0 | |
| **AMNH66448** |  | |  | | |  | |  | |  | |  | |  |  | |
| **M1** | 0 | | 0 | | | 0 | | 1 (88%) | | 0 | | 4 (440%) | |  | 0 | |
| **M2** | 1 (57%) | | 0 | | | 0 | | 5 (286%) | | 0 | | 0 | |  | 1 (95%) | |
| **M3** | 0 | | 0 | | | 0 | | 0 | | 0 | | 1 (200%) | |  | 2 (533%) | |
| **M4** | 1 (88%) | | 2 (126%) | | | 1 (147%) | | 0 | | 0 | | 0 | |  | 1 (147%) | |
| **M5** | 0 | | 0 | | | 0 | | 0 | | 0 | | 0 | |  | 0 | |
| **M6** | 1 (115%) | | 0 | | | 1 (192%) | | 0 | | 0 | | 0 | |  | 2 (383%) | |
| **Tricuspid** | 0 | | 1 (114%) | | | 0 | | 0 | | 0 | | 2 (400%) | |  | 0 | |
| **AMNH66433** |  | |  | | |  | |  | |  | |  | |  |  | |
| **M1** | 0 | | 0 | | | 1 (32%) | | 14 (130%) | | 12 (130%) | |  | |  |  | |
| **M2** | 0 | | 0 | | | 1 (285%) | | 0 | | 4 (380%) | |  | |  |  | |
| **M3** | 1 (54%) | | 0 | | | 0 | | 1 (104%) | | 2 (242%) | |  | |  |  | |
| **M4** | 13 (165%) | | 0 | | | 0 | | 0 | | 1 (29%) | |  | |  |  | |
| **M5** | 6 (93%) | | 2 (167%) | | | 3 (313%) | | 1 (30%) | | 0 | |  | |  |  | |
| **AMNH66434** |  | |  | | |  | |  | |  | |  | |  |  | |
| **M1** | 0 | | 0 | | |  | | 9 (248%) | | 1 (28%) | | 0 | |  |  | |
| **M2** | 0 | | 0 | | |  | | 0 | | 6 (308%) | | 0 | |  |  | |
| **M4** | 7 (181%) | | 0 | | |  | | 0 | | 2 (43%) | | 3 (258%) | |  |  | |
| **M5** | 3 (78%) | | 8 (344%) | | |  | | 1 (22%) | | 0 | | 0 | |  |  | |
| **M6** | 0 | | 0 | | |  | | 3 (333%) | | 0 | | 0 | |  |  | |
| **AMNH64405** |  | |  | | |  | |  | |  | |  | |  |  | |
| **M1** | 0 | | 3 (304%) | | |  | | 0 | | 4 (101%) | |  | |  |  | |
| **M2** | 2 (134%) | | 0 | | |  | | 0 | | 1 (39%) | |  | |  |  | |
| **M4** | 1 (59%) | | 0 | | |  | | 0 | | 4 (137%) | |  | |  |  | |
| **M5** | 6 (508%) | | 1 (198%) | | |  | | 5 (593%) | | 0 | |  | |  |  | |
| **AMNH66351** |  | |  | | |  | |  | |  | |  | |  |  | |
| **M2** |  | | 0 | | | 1 (48%) | |  | | 8 (116%) | |  | |  |  | |
| **M3** |  | | 1 (70%) | | | 0 | |  | | 2 (127%) | |  | |  |  | |
| **M5** |  | | 5 (67%) | | | 5 (200%) | |  | | 0 | |  | |  |  | |
|  |  | |  | | |  | |  | |  | |  | |  |  | |


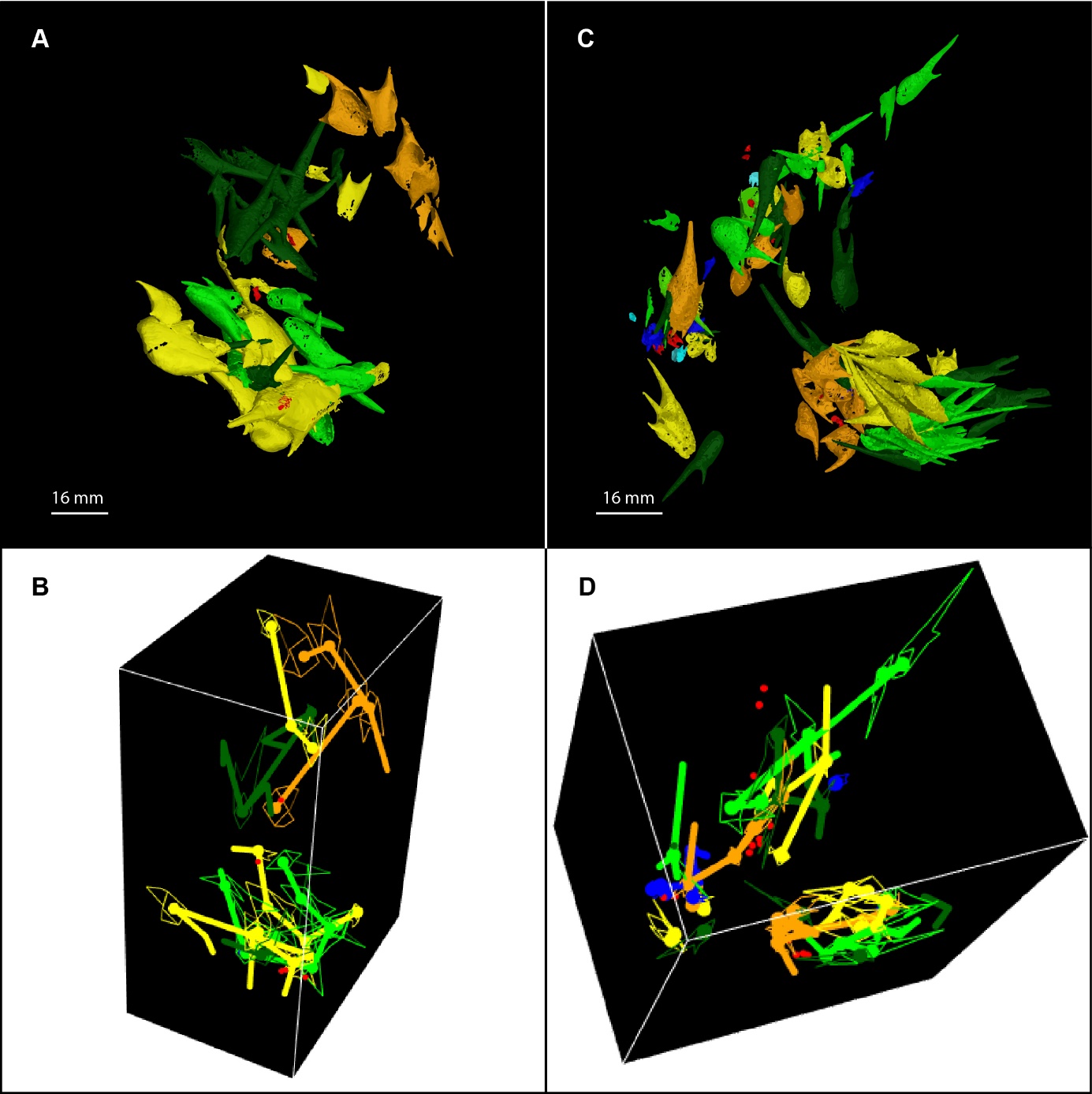


Figure S2**.** Distribution of the hooks *in-situ* **A & B –**AMNH 66434. **C & D –**AMNH 95795. **A & C –** 3D rendering (VGStudio MAX 3.0.) of the structures preserved in the body chamber. **B & D–** Simplified representation of the distribution of the hooks in space. The thick lines represent the links between the hooks according to morphotype, based on the persistent homology analysis of the centroid position of their openings. Only the strongest and best integrated links are shown. An additional animated figure is available in the corresponding supplementary “.gif” document and an interactive plot is available in the corresponding supplementary “.html” document.

**
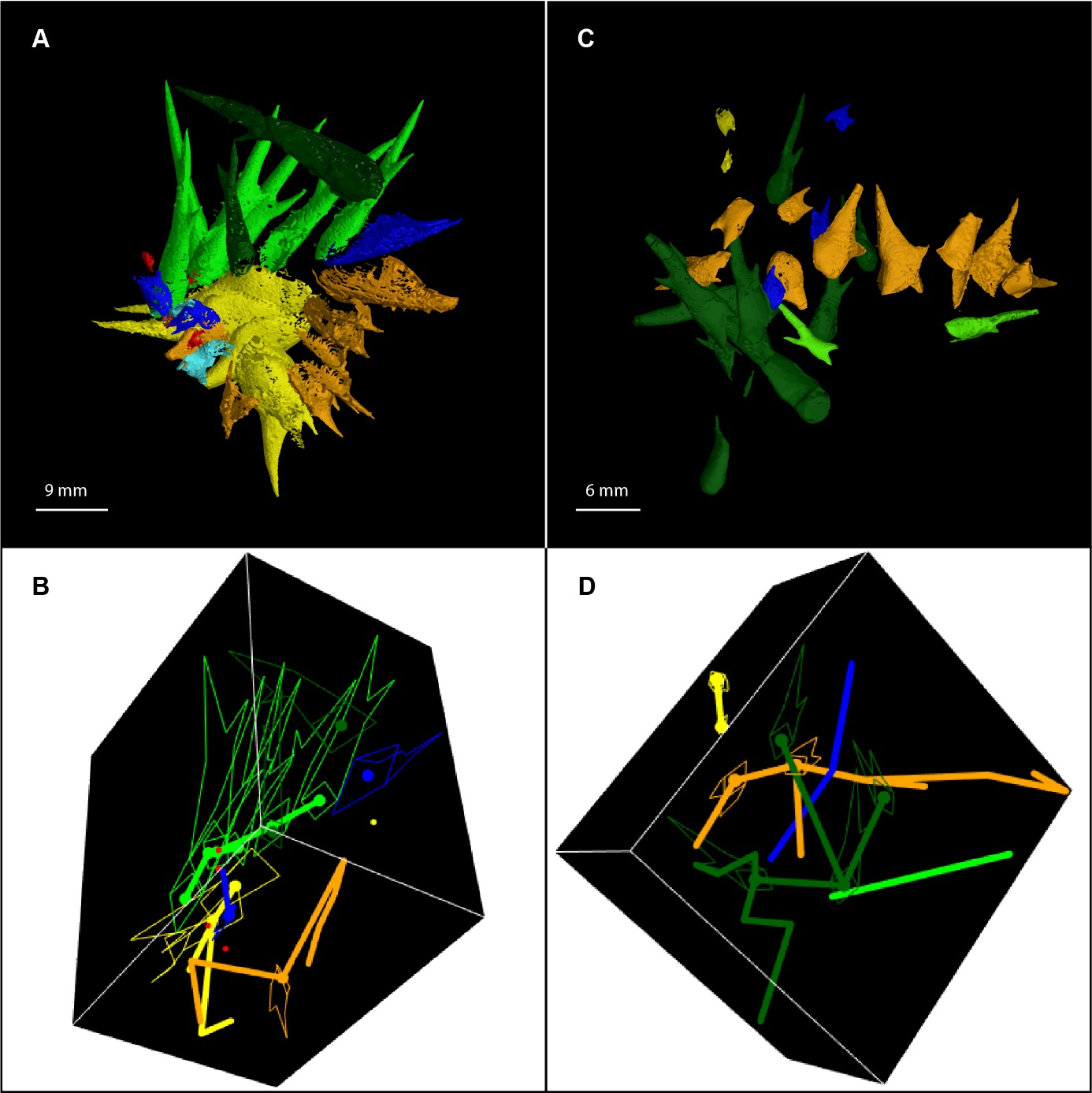
**

Figure S3**.** Distribution of the hooks *in-situ* **A & B –** AMNH 66448. **C & D –**AMNH 66351. **A & C –** 3D rendering (VGStudio MAX 3.0.) of the structures preserved in the body chamber. **B & D–** Simplified representation of the distribution of the hooks in space. The thick lines represent the links between the hooks according to morphotype, based on the persistent homology analysis of the centroid position of their openings. Only the strongest and best integrated links are shown. An additional animated figure is available in the corresponding supplementary “.gif” document and an interactive plot is available in the corresponding supplementary “.html” document.

**
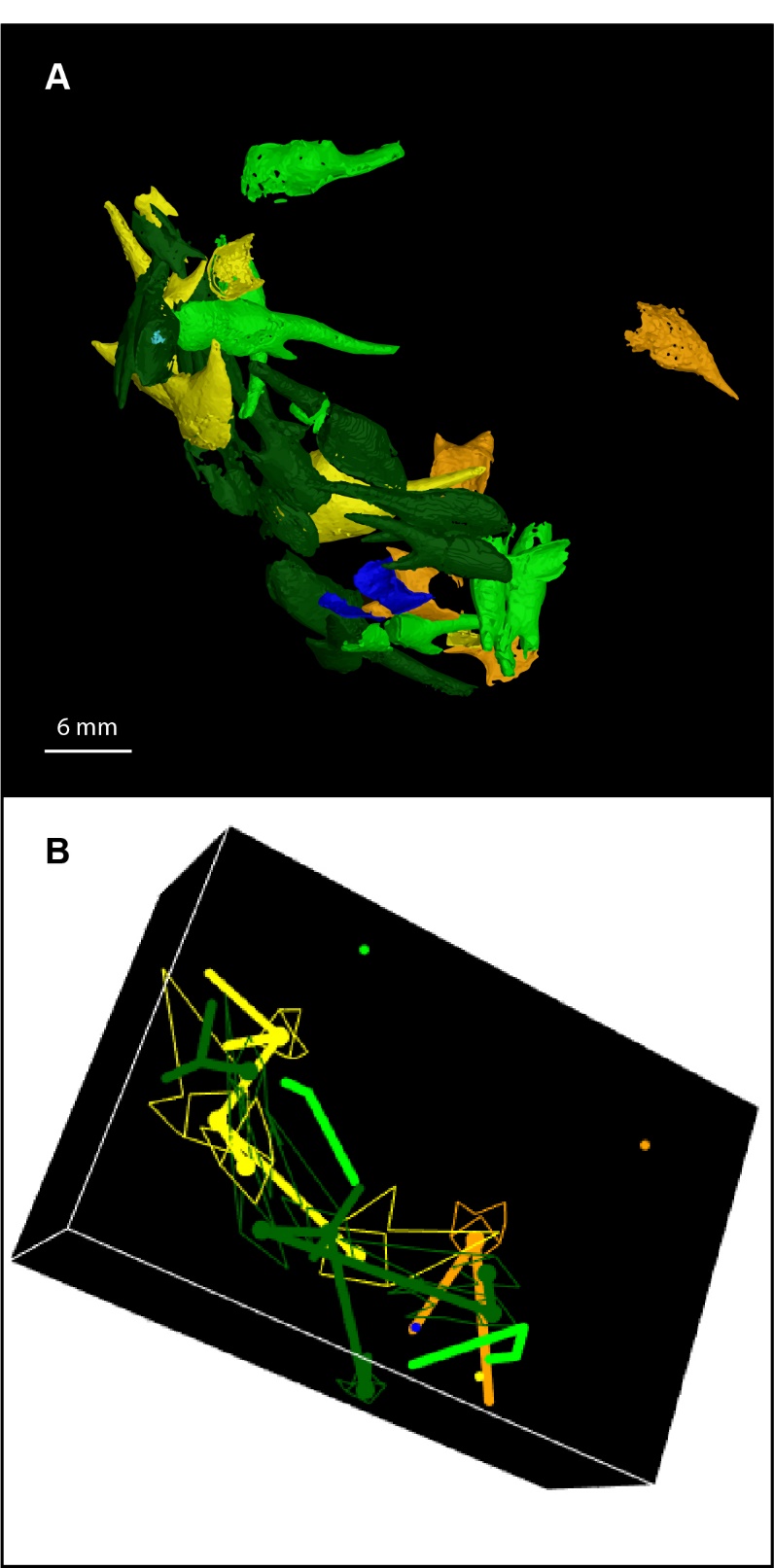
**

Figure S4**.** Distribution of the hooks *in-situ* in AMNH 64405. **A–** 3D rendering (VGStudio MAX 3.0.) of the structures preserved in the body chamber. **B–** Simplified representation of the distribution of the hooks in space. The thick lines represent the links between the hooks according to morphotype, based on the persistent homology analysis of the centroid position of their openings. Only the strongest and best integrated links are shown. An additional animated figure is available in the corresponding supplementary “.gif” document and an interactive plot is available in the corresponding supplementary “.html” document.


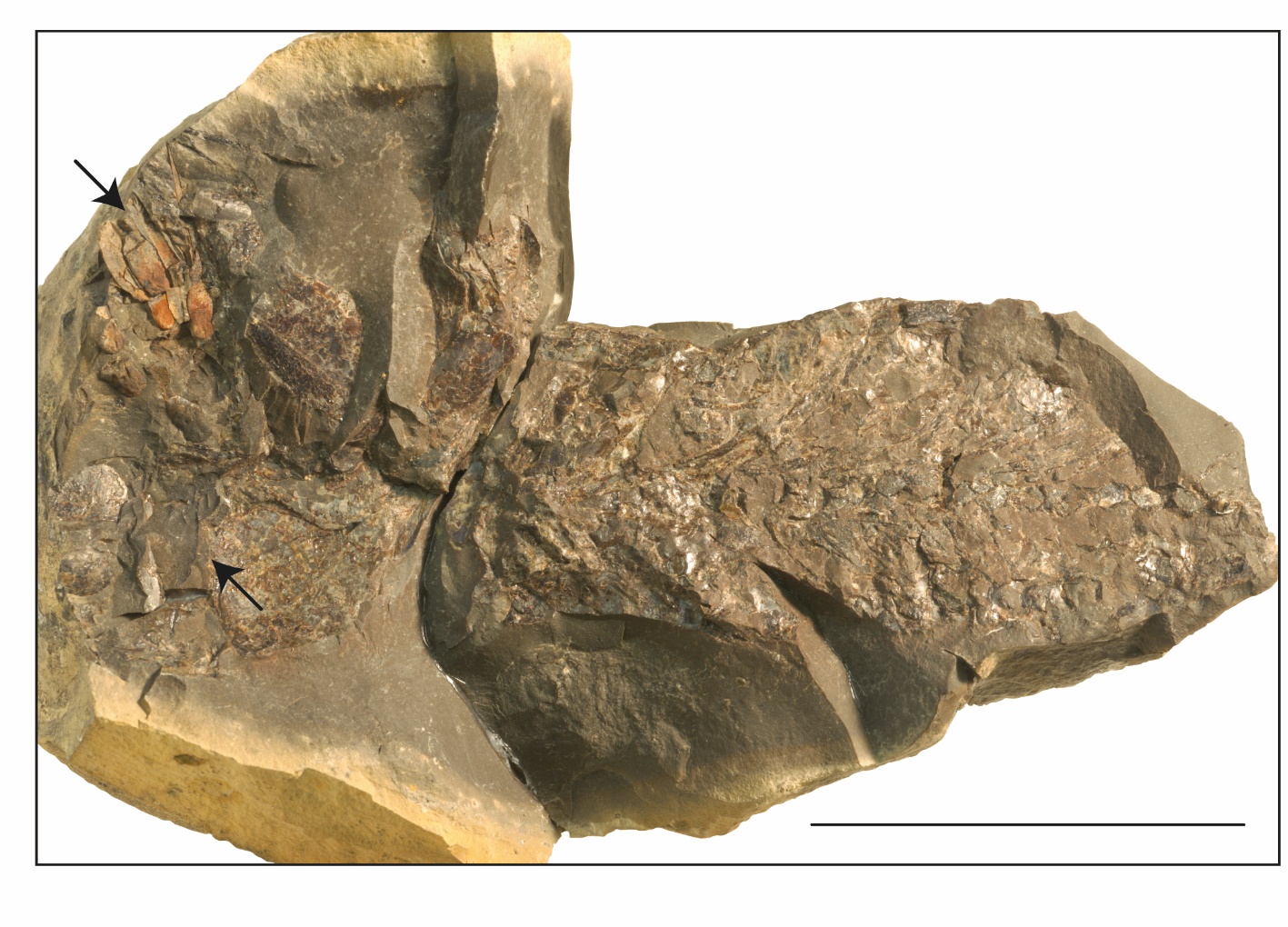
Figure S5. Concretion containing both fish remains and hooks (AMNH 108504). The hooks are indicated by arrows. The scale is 5 cm.

# Figure caption for complementary animated and interactive plots:

**Supplementary material for Figure 3-B-Animated plot AMNH66350:** Animated simplified representation of the distribution of the hooks in space for AMNH66350 (R software, package rgl). The thick lines represent the links between the hooks according to morphotype, based on the persistent homology analysis of the centroid position of their opening. Only the strongest and best integrated links are shown. M1-clear green; M2-Orange; M3-Dark blue; M4-Yellow; M5-Dark green; M6-Red.

**Supplementary material for Figure 3-B-Interactive plot AMNH66350:** Interactive plot of the distribution of the hooks in space for AMNH66350 (R software, package rgl). The thick lines represent the links between the hooks according to morphotype, based on the persistent homology analysis of the centroid position of their opening. Only the strongest and best integrated links are shown. M1-clear green; M2-Orange; M3-Dark blue; M4-Yellow; M5-Dark green; M6-Red.

**Supplementary material for Figure 3-D-Animated plot AMNH66433:** Animated simplified representation of the distribution of the hooks in space for AMNH66433 (R software, package rgl). The thick lines represent the links between the hooks according to morphotype, based on the persistent homology analysis of the centroid position of their opening. Only the strongest and best integrated links are shown. M1-clear green; M2-Orange; M3-Dark blue; M4-Yellow; M5-Dark green; M6-Red.

**Supplementary material for Figure 3-D-Interactive plot AMNH66433:** Interactive plot of the distribution of the hooks in space for AMNH66433 (R software, package rgl). The thick lines represent the links between the hooks according to morphotype, based on the persistent homology analysis of the centroid position of their opening. Only the strongest and best integrated links are shown. M1-clear green; M2-Orange; M3-Dark blue; M4-Yellow; M5-Dark green; M6-Red.

**Supplementary material for Figure S2-B-Animated plot AMNH66434:** Animated simplified representation of the distribution of the hooks in space for AMNH66434 (R software, package rgl). The thick lines represent the links between the hooks according to morphotype, based on the persistent homology analysis of the centroid position of their opening. Only the strongest and best integrated links are shown. M1-clear green; M2-Orange; M4-Yellow; M5-Dark green; M6-Red.

**Supplementary material for Figure S2-B-Interactive plot AMNH66434:** Interactive plot of the distribution of the hooks in space for AMNH66434 (R software, package rgl). The thick lines represent the links between the hooks according to morphotype, based on the persistent homology analysis of the centroid position of their opening. Only the strongest and best integrated links are shown. M1-clear green; M2-Orange; M4-Yellow; M5-Dark green; M6-Red.

**Supplementary material for Figure S2-D-Animated plot AMNH95795:** Animated simplified representation of the distribution of the hooks in space for AMNH95795 (R software, package rgl). The thick lines represent the links between the hooks according to morphotype, based on the persistent homology analysis of the centroid position of their opening. Only the strongest and best integrated links are shown. M1-clear green; M2-Orange; M3-Dark blue; M4-Yellow; M5-Dark green; M6-Red.

**Supplementary material for Figure S2-D-Interactive plot AMNH95795:** Interactive plot of the distribution of the hooks in space for AMNH95795 (R software, package rgl). The thick lines represent the links between the hooks according to morphotype, based on the persistent homology analysis of the centroid position of their opening. Only the strongest and best integrated links are shown. M1-clear green; M2-Orange; M3-Dark blue; M4-Yellow; M5-Dark green; M6-Red.

**Supplementary material for Figure S3-B-Animated plot AMNH66448:** Animated simplified representation of the distribution of the hooks in space for AMNH66448 (R software, package rgl). The thick lines represent the links between the hooks according to morphotype, based on the persistent homology analysis of the centroid position of their opening. Only the strongest and best integrated links are shown. M1-clear green; M2-Orange; M3-Dark blue; M4-Yellow; M5-Dark green; M6-Red.

**Supplementary material for Figure S3-B-Interactive plot AMNH66448:** Interactive plot of the distribution of the hooks in space for AMNH6644 (R software, package rgl)8. The thick lines represent the links between the hooks according to morphotype, based on the persistent homology analysis of the centroid position of their opening. Only the strongest and best integrated links are shown. M1-clear green; M2-Orange; M3-Dark blue; M4-Yellow; M5-Dark green; M6-Red.

**Supplementary material for Figure S3-D-Animated plot AMNH66351:** Animated simplified representation of the distribution of the hooks in space for AMNH66351 (R software, package rgl). The thick lines represent the links between the hooks according to morphotype, based on the persistent homology analysis of the centroid position of their opening. Only the strongest and best integrated links are shown. M1-clear green; M2-Orange; M3-Dark blue; M4-Yellow; M5-Dark green.

**Supplementary material for Figure S3-D-Interactive plot AMNH66351:** Interactive plot of the distribution of the hooks in space for AMNH66351 (R software, package rgl). The thick lines represent the links between the hooks according to morphotype, based on the persistent homology analysis of the centroid position of their opening. Only the strongest and best integrated links are shown. M1-clear green; M2-Orange; M3-Dark blue; M4-Yellow; M5-Dark green.

**Supplementary material for Figure S4-B-Animated plot AMNH66405:** Animated simplified representation of the distribution of the hooks in space for AMNH66405 (R software, package rgl). The thick lines represent the links between the hooks according to morphotype, based on the persistent homology analysis of the centroid position of their opening. Only the strongest and best integrated links are shown. M1-clear green; M2-Orange; M3-Dark blue; M4-Yellow; M5-Dark green.

**Supplementary material for Figure S4-B-Interactive plot AMNH66405:** Interactive plot of the distribution of the hooks in space for AMNH66405 (R software, package rgl). The thick lines represent the links between the hooks according to morphotype, based on the persistent homology analysis of the centroid position of their opening. Only the strongest and best integrated links are shown. M1-clear green; M2-Orange; M3-Dark blue; M4-Yellow; M5-Dark green.
